# Nanohoops in membranes: confined supramolecular spaces within phospholipid bilayer membranes†

**DOI:** 10.1039/d4sc03408b

**Published:** 2024-09-11

**Authors:** Kylie Chinner, Niklas Grabicki, Rei Hamaguchi, Mitsunori Ikeguchi, Kazushi Kinbara, Sayaka Toyoda, Kohei Sato, Oliver Dumele

**Affiliations:** a Department of Chemistry and IRIS Adlershof, Humboldt-Universität zu Berlin Brook-Taylor-Str. 2 Berlin 12489 Germany; b School of Life Science and Technology, Tokyo Institute of Technology Yokohama Kanagawa 226-8501 Japan; c Graduate School of Medical Life Science, Yokohama City University Yokohama Kanagawa 230-0045 Japan; d Research Center for Autonomous SystemMaterialogy (ASMat), Institute of Innovative Research, Tokyo Institute of Technology Kanagawa 226-8501 Japan; e Department of Chemistry, School of Science 1 Gakuen Uegahara Sanda-shi Hyogo 669-1330 Japan ksato@kwansei.ac.jp https://www.ksatolab.net; f Institute of Organic Chemistry, University of Freiburg Albertstr. 21 Freiburg 79104 Germany oliver.dumele@oc.uni-freiburg.de https://www.dumelelab.com

## Abstract

Nanohoops, an exciting class of fluorophores with supramolecular binding abilities, have the potential to become innovative tools within biological imaging and sensing. Given the biological importance of cell membranes, incorporation of macrocyclic materials with the dual capability of fluorescence emission and supramolecular complexation would be particularly interesting. A series of different-sized nanohoops—ethylene glycol-decorated [*n*]cyclo-*para*-pyrenylenes (CPYs) (*n* = 4–8)—were synthesised *via* an alternate synthetic route which implements a stannylation-based precursor, producing purer material than the previous borylation approach, enabling the growth of single-crystals of the Pt-macrocycle. Reductive elimination of these single-crystals achieved significantly higher selectivity and yields towards smaller ring-sized nanohoops (*n* = 4–6). The supramolecular binding capabilities of these CPYs were then explored through host–guest studies with a series of polycyclic (aromatic)hydrocarbons, revealing the importance of molecular size, shape, and CH–π contacts for efficient binding. CPYs were incorporated within the hydrophobic layer of lipid bilayer membranes, as confirmed by microscopic imaging and emission spectroscopy, which also demonstrated the size-preferential incorporation of the five-fold nanohoop. Molecular dynamics simulations revealed the position and orientation within the membrane, as well as the unique non-covalent threading interaction between nanohoop and phospholipid.

## Abstract 2

Strained ethylene glycol-decorated nanohoops as biological fluorophores were synthesised with improved efficiency and their host–guest bindings were explored. Incorporation into lipid bilayers showcases the potential for biological applications.

## Graphical abstract

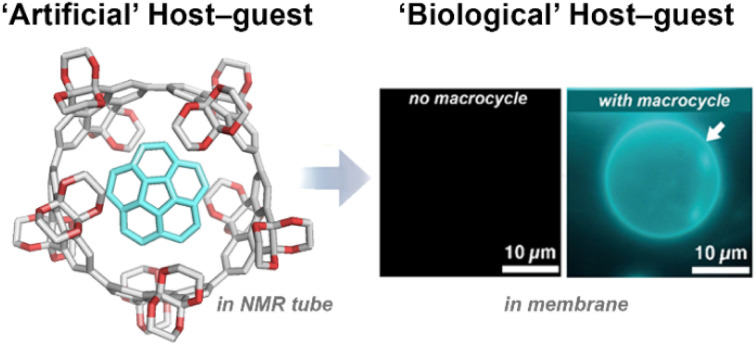

## Introduction

Carbon-rich nanohoops have emerged as intriguing structures since their first synthesis in 2008,^1^ captivating researchers with their innovative synthetic aspects,^2,3^ unique optoelectrical properties,^4,5^ and potential for supramolecular chemistry.^6–8^ These nanohoops are finite macrocyclic segments of carbon nanotubes:^9^ hollow sp^2^ cylindrical structures which possess high tensile strength, thermal conductivity, and the ability to be semiconducting or metallic depending on the orientation of their lattice.^10,11^ The radially cyclic π-systems of these inherently strained aromatic rings can produce size-dependent photophysical properties such as fluorescence emission—unique when compared to their non-cyclised counterparts.^12–14^ These properties can be altered further with the introduction of heteroatoms or polycyclic aromatic moieties.^15–20^ Additionally, inherent shape-persistence and inner π-surface of nanohoops make these rings ideal hosts for host–guest interactions^21–26^ and the formation of supramolecular assemblies.^6,7^

Recently, carbon-rich nanohoops as an emerging class of biological fluorophores for sensing and imaging have been of particular interest,^27–30^ some recent examples being the aqueous-soluble sulfonated [8]cycloparaphenylene which can penetrate live cells^27^ and [10]cycloparaphenylene assembled into cell-incorporated nanoscale vesicles.^29^ While nanohoops within membranes have been previously studied for bioimaging and sensing applications, their unique supramolecular complexation capabilities within membranes has yet to be exploited. Additionally, nanohoops have large Stokes shifts (110–250 nm), with no spectral overlap between absorbance and emission.^12^ This means, in principle, a mixture of ring sizes can all be excited at the same wavelength, resulting in a size-dependent readout, facilitating localisation.

Previously, we reported that amphiphilic molecules can be effectively incorporated into lipid bilayer membranes^31–37^ to form intriguing supramolecular structures such as reversible calcium-induced assemblies,^38^ or self-assembled supramolecular transmembrane ion channels.^36^

Based on those findings, the advantageous fluorescence emission of nanohoops,^39^ paired with their shape-persistence^40^ and defined inner void with guest binding capabilities, we envisioned that functionalised cycloparaphenylene-type nanohoops would exhibit unique properties when incorporated into artificial and natural membranes.

While several methods have been established to access the shortest possible repeating units of carbon nanotubes, called cycloparaphenylenes,^41–47^ applications remain in their infancy due to synthetic challenges imposed by the high energy required to bend an aromatic ring out of plane. Larger nanorings^48,49^—such as Isobe and co-workers’ phenine nanoring^50,51^—avoid this, achieving an almost strain-free macrocycle through a considerably wider diameter. While larger nanorings can undergo host–guest binding, prominently with fullerene, efficient binding of non-fullerene guests to nanohoops with smaller diameters is still rare.^52,61–66^

Recently, we reported the synthesis of a series of highly functionalised nanohoops, [*n*]cyclo-2,7-(4,5,9,10-tetrahydro)pyrenylenes (CPYs),^52^ following the platinum-mediated macrocyclisation developed by Yamago and co-workers.^54^ These nanohoops featured ethylene glycol moieties which decorate the outer rim and confine the inner cavity. The five-fold CPY in particular, possesses high fluorescence emission and an optimal sized cavity for small molecule binding, making it an excellent shape-persistent candidate to study supramolecular host–guest interactions. However, utilising the reversible Pt-complexation often results in complex product mixtures, typically containing a range of ring sizes of reductively eliminated carbon nanorings. ^43,44,52^ Thus, additional selectivity is desired.

**Fig. 1:**
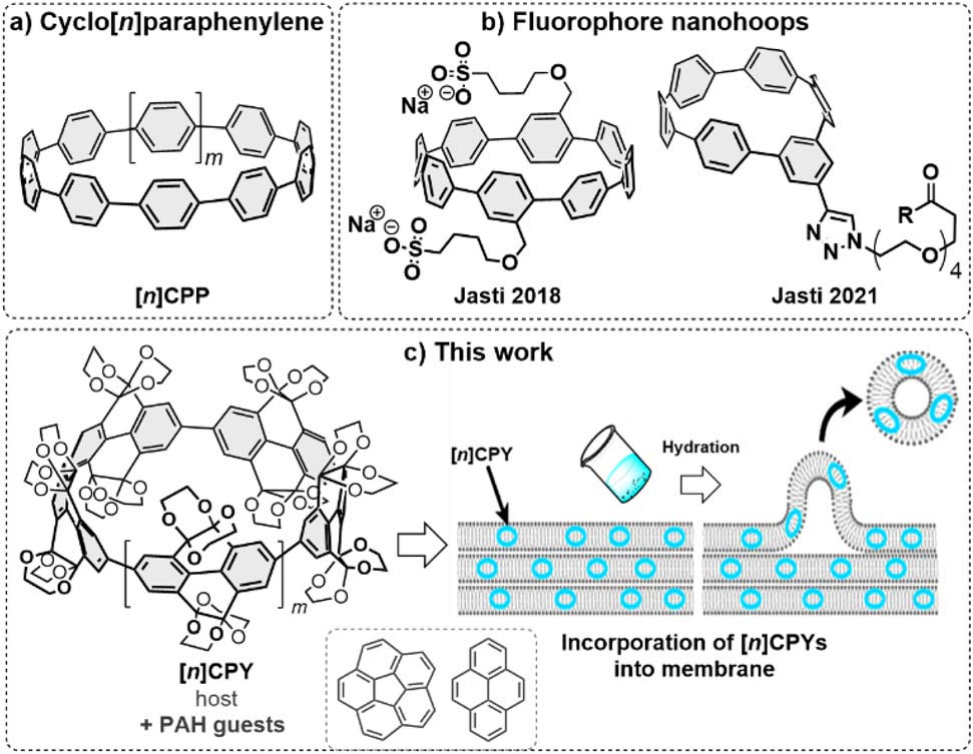
(a) [*n*]Cycloparaphenylenes ([*n*]CPP) and (b) reported [*n*]CPP derivatives as biological nanohoop fluorophores.^27,28^ (c) [*n*]CPYs as biological nanohoop fluorophores for host–guest complexation with polycyclic aromatic hydrocarbon (PAH) guests and incorporation into the hydrophobic layer of phospholipid bilayer membranes.

Herein, we report a size-selective route towards CPYs with higher overall yields and ring size selectivity (*n* = 4–6), with the reductive elimination on isolated single-crystalline Pt-macrocycle as a key step. With this new-found control in synthesis over size to achieve precise molecular entities, we took advantage of the size distribution and identified the five-fold as the most favourable CPY—regarding its high fluorescence emission and optimal central void volume—for encapsulation of non-polar hydrocarbon guests and selective incorporation into phospholipid bilayer membranes (Fig. 1).^55^

## Results and discussion

### A size-selective route towards smaller ring-sized nanohoops

**Scheme 1:**
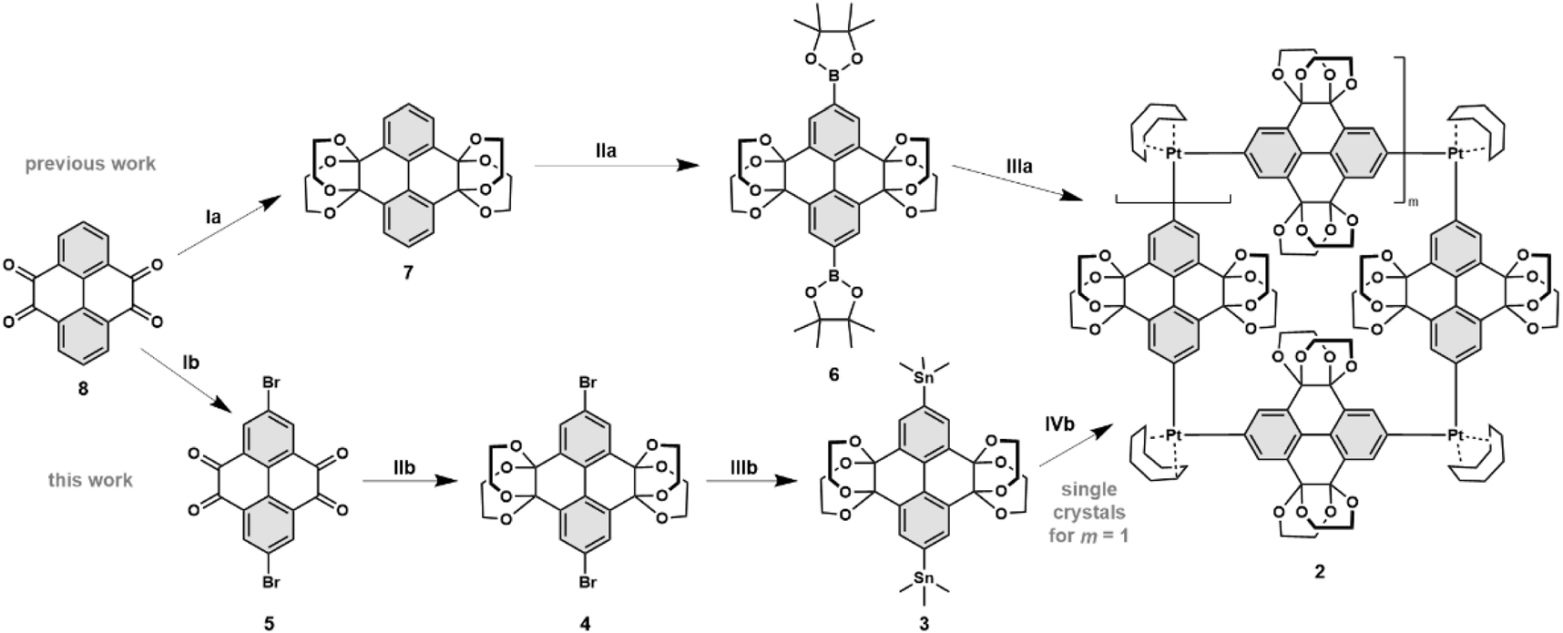
Synthesis of 2 as previously synthesised^52^ starting from 2,7-bisbromo-pyrene-4,5,9,10-tetrone 5. Reagents and conditions: (Ia) camphorsulfonic acid, ethylene glycol, MeOH, 120 °C, 24 h, 73%; (IIa) 4,4′-di-*tert*-butyl-2,2′-bipyridine, [Ir(OMe)(COD)]_2_, bis(pinacolato)diboron, 1,4-dioxane, 120 °C, 18 h, 84%; (IIIa) [PtCl_2_(COD)], CsF, CH_2_Cl_2_, 45 °C, 24 h, 74%. Synthesis of 2 as single-crystals starting from 2,7-bisbromo-pyrene-4,5,9,10-tetrone 5. Reagents and conditions: (Ib) camphorsulfonic acid, ethylene glycol, MeOH, 120 °C, 48 h, 70%; (IIb) Pd(PPh_3_)_4_, LiCl, 2,4,6-tri-*tert*-butylphenol, (Sn(Me_3_))_2_, 1,4-dioxane, 110 °C, 2 h, 77%; (IIIb) Pt(COD)Cl_2_, THF, 60 °C, 24 h, 74%.

Our previous synthetic route^52^ towards highly functionalised CPYs used a bisboronic precursor for the platinum mediated macrocyclization (Scheme 1, top), which resulted in a mixture of reductively eliminated carbon nanorings of various ring sizes (*n* = 4–8).^51–61^ While the square-planar geometry of platinum(ii) complexes should dictate a four-fold ring, due to the sterically demanding ethylene glycol groups and certain flexibility in regard to the angle between the platinum ligands, higher ring-sizes are also formed.^52^ The platinum aryl bond is reversible under certain conditions, dependent on the ligand and the solvent.^62^ We assumed that it could be possible to selectively crystallise the four-fold Pt-metallacycle, however we were not able to obtain any crystalline product from Pt-metallacycle mixtures obtained *via* a bisboronic precursor.^52^ To eliminate potential impurities preventing the crystallisation process, we aimed to explore an alternative precursor bearing trimethylstannyl groups.^57^

**Fig. 2:**
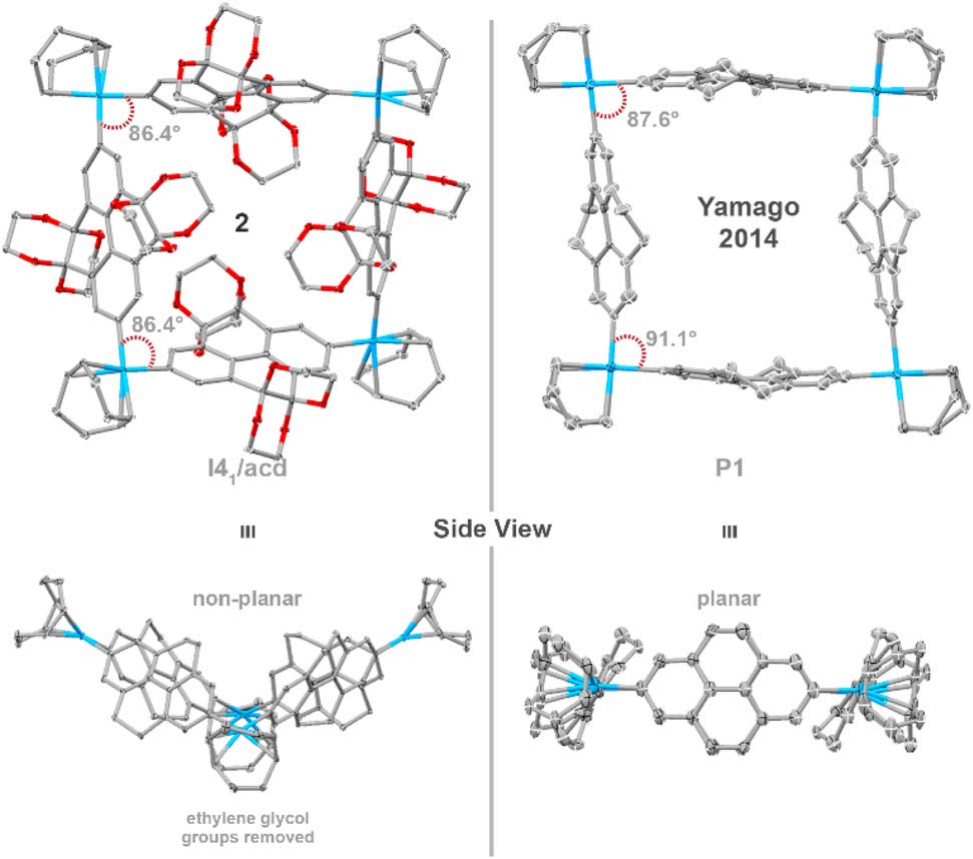
Single-crystal X-ray structure of platinum macrocycle 2 with side view and Yamago and co-workers’ 2014 platinum macrocycle^44^ for comparison. Ellipsoids are shown at 50% probability; hydrogen atoms and solvent molecules are omitted for clarity. Colour code: carbon, grey; oxygen, red.

To obtain bisstannane precursor 3, the *K*-region of pyrene was symmetrically functionalised *via* a Ru-catalysed oxidation to obtain pyrene-4,5,9,10-tetrone 6 as previously reported (see ESI Scheme S1†).^52^ Bromination with NBS at the 2 and 7 positions gave 5, followed by an acid-catalysed condensation with ethylene glycol to afford 4 (Scheme 1, bottom). Next, Pd-catalysed stannylation gave the 2,7-bis(trimethylstannyl)pyrene precursor 3, then subsequent transmetalation with an equimolar amount of dichloro(1,5-cyclooctadiene)platinum(ii) produced the macrocyclic Pt(ii) precursor 2 in 74% yield as an off-white solid that crystallised upon slow evaporation from a saturated solution in CH_2_Cl_2_, resulting in crystals suitable for X-ray diffraction (Fig. 2).

Interestingly, this platinum macrocycle differs in geometry from Yamago and co-worker's platinum macrocycle.^44^ While both are comprised of a repeating pyrene scaffold, introduction of sterically demanding ethylene glycol motifs distort the nanoring from planarity to a non-planar ‘butterfly’ geometry—as revealed by the C_Ar_–Pt(ii)–C_Ar_ angles of 86.4°, which are slightly smaller than those of Yamago and co-workers’ 4,5,9,10-tetrahydropyrenylene-containing platinum metallacycle (91.1° and 87.6°, Fig. 2).

**Fig. 3:**
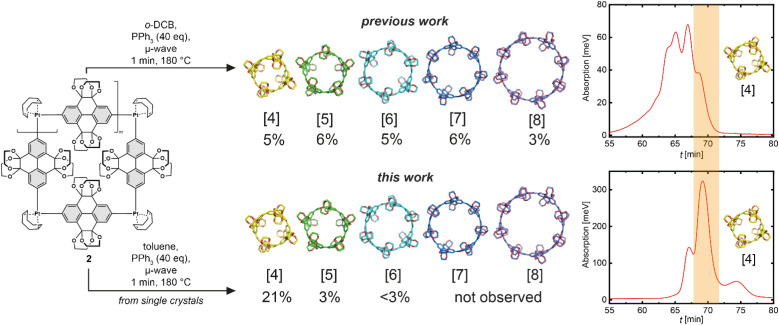
Comparison of the reductive elimination from a sample containing different ring sizes of the platinum metallacycle (previous work) and a sample of single-crystals of the four membered metallacycle (this work), with corresponding yields and the rGPC chromatograms of the crude reaction mixture.

Reductive elimination on single-crystals of the platinum metallacycle precursor 2 gave the targeted CPYs 1_[_*_n_*_]_ with unprecedented size-selectivity of 1_[4]_/1_[5]_/1_[6]_ 7 : 1 : <1. This is a significant increase in ring size selectivity compared to the reductive elimination from the typical crude Pt-metallacycle mixture, which was previously reported to 1_[4]_/1_[5]_/1_[6]_/1_[7]_/1_[8]_ 2 : 2 : 2 : 2 : 1, and an overall improved yield for the preferred smaller ring sizes (from 5% to 21% for 1_[4]_). Separation of ring sizes by recycling gel permeation chromatography (rGPC) unambiguously illustrates this selectivity, with a significantly larger peak area permeating for the four-fold ring, 1_[4]_, after reductive elimination of single-crystals (Fig. 3, right). The residual larger ring sizes *n* > 4 originate from either small impurities within the single-crystalline material containing larger platinum metallacycles, or from Pt(PPh_3_)_4–*n*_ formation—which is known to insert into strain-activated C–C bonds—and subsequent ligand exchange.^59,60^ While the more selective synthesis of 1_[4]_ provides valuable insight into the reaction mechanism, this ring size remains of least importance compared to its larger congeners for guest binding due to the very small gate opening and inner cavity. Therefore, we turned to 1_[5]_ to explore new guest binding abilities to expand upon previously reported cationic crown–ether complexes.^52,53^

### Host–guest studies of 1_[5]_ with non-polar polycyclic hydrocarbons

**Fig. 4:**
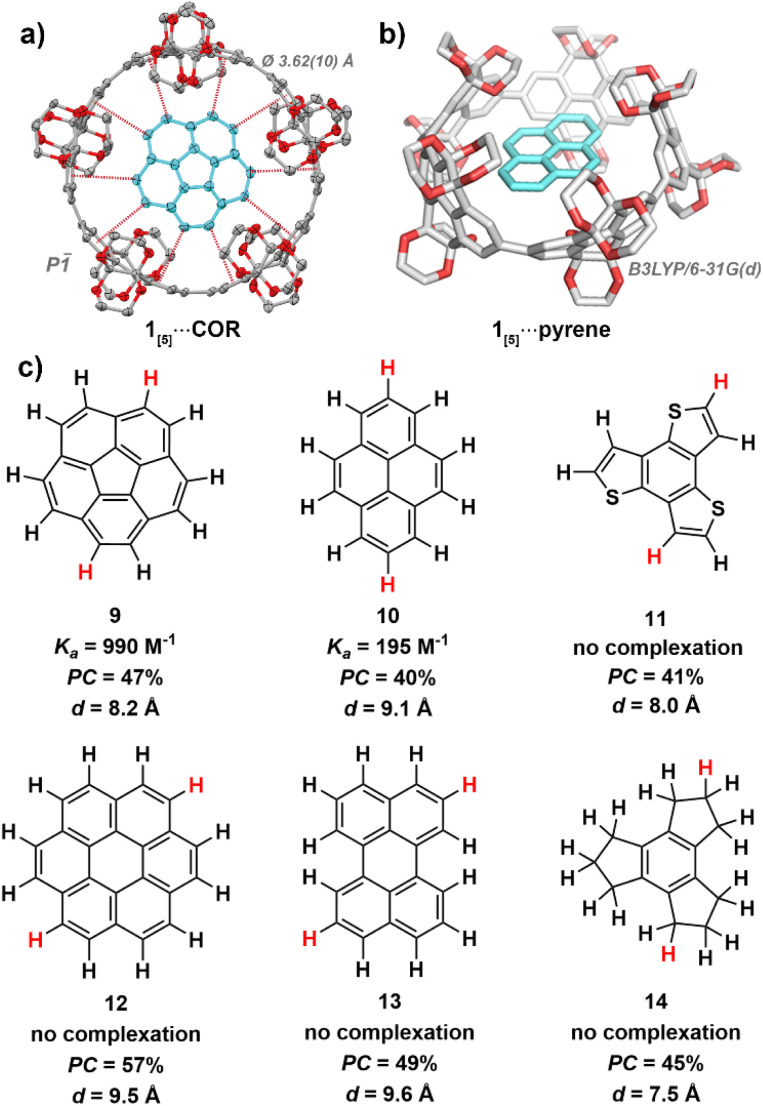
(a) X-ray crystal structure of 1_[5]_⋯COR (100 K). Ellipsoids are shown at 50% probability; hydrogen atoms and solvent molecules are omitted for clarity. Colour code: carbon, grey; oxygen; red. (b) Calculated geometry of 1_[5]_⋯pyrene, level of theory: DFT:B3LYP/6-31G(d). (c) Guest molecules used in host–guest studies with 1_[5]_. Association constants (*K*_a_) obtained *via*^1^H NMR titration experiments in benzene-*d*_6_ at 298 K (see ESI Fig. S19†). Packing coefficients (PC) obtained from the calculated cavity volumes of X-ray (1_[5_]) and geometry-optimised (guests) structures using the MS Roll suite implemented in X-Seed (see ESI Section S5†). Diameters (*d*) were derived from geometry-optimised structures, with the largest distance measured between two hydrogens taken (highlighted in red).

Previous studies have shown that shape-persistent [*n*]cycloparaphenylenes bind small aromatic molecules.^61–64,66^ Therefore, after finding the first example of complexation between CPYs and crown ethers,^52^ we began screening non-polar hydrocarbons as guests within the confined cavity of 1_[5]_—the five-fold nanohoop chosen specifically due to its favourable degree of confinement regarding the central void volume and high fluorescence emission (Fig. 4).

Through isothermal ^1^H NMR titrations at slow exchange, corannulene^67–70^ (9) was found to bind the strongest of all guests screened within this study, with an association constant of 990 M^−1^ in benzene-*d*_6_ at 298 K (see ESI Fig. S19†). The C_5_ symmetry of 1_[5]_ produces a favourable alignment for corannulene's (9) C_5V_ symmetric bowl-shape and 10 peripheral hydrogen atoms, leading to optimal average CH–π contact distance of *d*_C–π_ = 3.62(10) Å as satisfyingly revealed by single-crystal X-ray crystallography (see ESI Section S4.2†).

This is particularly intriguing as corannulene (9) is known to bind with smaller sized nanohoops, such as [10]cycloparaphenylene^66^ (*d*_[10]CPP_ = 13.8 Å, *K*_a_ = 170 ± 50 M^−1^ in CDCl_3_ at 293 K) and [4]cyclo-2,8-chrysenylene ([4]CC)^64^ (*d*_[4]CC_ = 13.4 Å, *K*_a_ = 2940 M^−1^ in CD_2_Cl_2_ at 298 K), whereas the cavity of 1_[5]_ should be too large (15.2 Å) for efficient binding. Nonetheless, binding does occur. Therefore, the highly functionalised rim of 1_[5]_ (gate diameter of 9.8 Å) must be a significant factor in enabling corannulene (9) to complex within the oversized inner cavity of 1_[5]_. Thus, the unique ethylene glycol-decorated rim of CPYs can enable binding with guests that would typically not occur due unfavourable size difference between host cavity and guest.

Pyrene (10), the second strongest bound hydrocarbon guest of the investigated series, had a significantly smaller association constant of 195 M^−1^ in benzene-*d*_6_ at 298 K (see ESI Fig. S20†). While both corannulene (9) and pyrene (10) possess 10 exposed hydrogen atoms, the hydrogens of pyrene (10) are arranged in an ovoid fashion. This ovaloid shape likely leads to suboptimal contact, and therefore weaker CH–π interactions between pyrene (10) and 1_[5]_. Additionally, while other strained carbon nanorings can undergo flexible alignment to gain optimal CH–π contacts around guest molecules, as seen with Isobe and co-workers’ [4]cyclo-2,8-chrysenylene,^64^ the bulky ethylene glycol groups of 1_[5]_ likely prevent this distortion due to steric hindrance.

As the cavity of 1_[5]_ is both confined and restricted from distortion by the highly functionalised rim, size and shape are crucial factors in host–guest complexation. Coronene (12), while possessing 12 hydrogen atoms, increasing the potential for CH–π interactions, is too large and reveals no evidence of binding upon mixing with 1_[5]_ in benzene-*d*_6_ at 298 K—as with perylene (13), which is too wide to fit within the confined cavity. Initially, this was surprising as coronene (12) is known to bind with [11]cycloparaphenylene^66^ (15.2 Å), which shares a similar inner cavity diameter as 1_[5]_ (15.2 Å). However, when considering the smaller gate diameter (9.8 Å) of 1_[5]_, facilitated by its highly functionalised rim, the lack of complexation becomes understandable.

Benzo[1,2-*b*:3,4-*b*′:5,6-*b*″]trithiophene (11), which possesses a similar diameter as corannulene (9), did not bind—possibly lacking the sufficient amount of C–H moieties required for measurable complex formation. We hypothesised that corannulene's (9) ability to undergo bowl inversion^72–75^ possibly allowed the fluctuating molecule to fit within the cavity despite its curved shape, hence we screened the partially unsaturated and more flexible 2,3,4,5,6,7,8,9-octahydro-1*H*-trindene (14). However, it did not bind—likely due weaker C_sp_3__H–π interactions and suboptimal contact angles from the less polarised alkane hydrogens which cannot point directly at the π-electrons of 1_[5]_.

Interestingly, changing solvents to chlorinated tetrachloroethane-*d*_2_ resulted in a lower association constant for corannulene (9) (622 M^−1^, compared to 990 M^−1^ in benzene-*d*_6_, both at 298 K) (see ESI Fig. S21†). While, unexpectedly, ^1^H NMR titrations in CDCl_3_ displayed no binding. A single-crystal X-ray structure of 1_[6]_ grown from CHCl_3_ shows competitive solvent interaction between multiple chloroform molecules and the oxygen atoms of the ethylene glycol moieties at the outer rim of the nanohoop's cavity, which reveal an enthalpically favourable solvate complex compared to non-polar hydrocarbon guest binding (see ESI Fig. S13c†).

### 1 : 1 Complexation of 1_[5]_ and corannulene

**Fig. 5:**
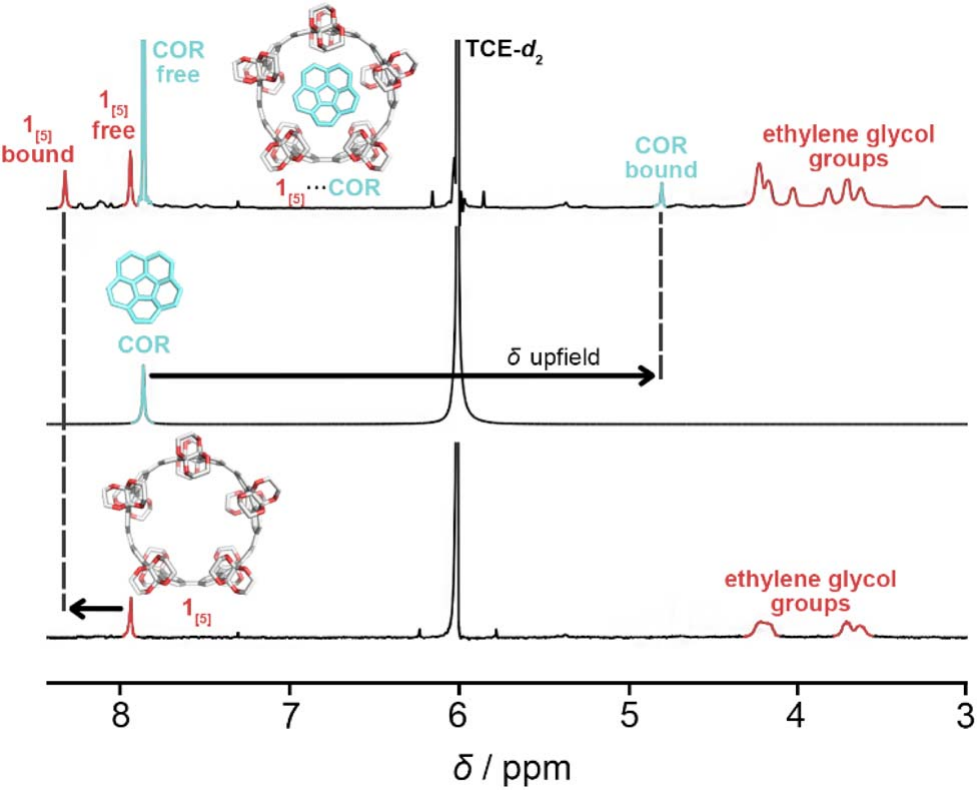
^1^H NMR spectra (600 MHz) for the assignment of the complexation of 1_[5]_ and corannulene (COR) in tetrachloroethane-*d*_2_ (TCE-*d*_2_) at 298 K.

The complexation of 1_[5]_ and corannulene (COR) in tetrachloroethane-*d*_2_ (298 K) was first observed *via*^1^H NMR titration through the emergence of two new singlets (Fig. 5). The dramatic upfield resonance shift of corannulene to 5.70 ppm and the downfield shift of 1_[5]_ to 8.16 ppm is consistent with shielding/deshielding effects of a bowl-in-tube structure of 1_[5]_⋯COR, similar to Isobe and co-workers’ [4]CC nanoring binding with corannulene.^23^ The presence of four singlets—‘free’ and ‘bound’ of both 1_[5]_ and corannulene—upon titration is ascribed to a slow in-and-out exchange process.

**Fig. 6:**
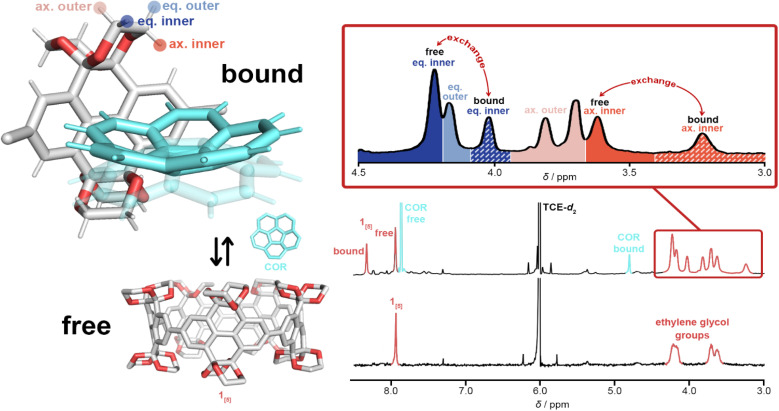
^1^H NMR spectra (C_2_D_2_Cl_4_, 600 MHz, 298 K) illustrating the assignment of ethylene glycol groups of 1_[5]_⋯COR with a schematic complexation of free 1_[5]_, free corannulene, and the complex 1_[5]_⋯COR. Only one wall fragment of 1_[5]_ in the complex is illustrated for clarity. Solid-convex and transparent-concave corannulene are shown as a representation of corannulene's bowl inversion. Assigned *via*^1^H–^1^H ROESY and ^1^H–^1^H NOESY spectra with key NOE cross-signals marked as red arrows (for further details, see ESI Fig. S22 and S23†).

For uncomplexed 1_[5]_, two broad ^1^H NMR signals corresponding to the ethylene glycol moieties (*ca.* 4.25 and 3.70 ppm) are characteristic, with the equatorial protons shifted further downfield than the axial protons due to hyperconjugation with oxygen.^71^ The pseudo doublet of the axial protons is typical of the smaller ring sizes of CPYs (1_[4]_ and 1_[5]_).^52^ However, upon guest addition, the ^1^H NMR signals of the ethylene glycol groups in 1_[5]_ began to split (Fig. 6) (see ESI Fig. S21†). As both equatorial and axial inner protons of the ethylene glycol moiety face the inner cavity of 1_[5]_, they can be influenced by the presence of a guest. Consequently, for the complexation of 1_[5]_⋯COR, the ^1^H NMR signals of ‘free’ and ‘bound’ ethylene glycol split further into ‘bound’ and ‘free’ equatorial inner protons (H_eq. inner_) as well as ‘free’ and ‘bound’ axial inner (H_ax. inner_) (these signals were identified and assigned *via*^1^H–^1^H ROESY and ^1^H–^1^H NOESY experiments, see ESI Fig. S22 and S23†). The ethylene glycol protons facing away from the cavity (H_eq. outer_ and H_ax. outer_) maintain similar chemical shifts to the uncomplexed host, with the same pseudo doublet of the axial protons present. A relative population of free/bound COR in 1_[5]_⋯COR was found to be 38 : 62 at 8 equivalents of corannulene at 298 K (see ESI Fig. S21†).

Additionally, corannulene is known to undergo degenerate bowl-to-bowl inversion^72–75^ which passes through a planar D_5h_ transition state. As 1_[5]_ can supply a distinct chemical environment through the ‘inner’ and ‘outer’ protons of its ethylene glycol decorated rim, we hypothesised that the unique splitting pattern of our host, paired with the increased stability gained from host–guest complexation, could reveal the bowl inversion of nascent corannulene at low temperatures, similar to that of Exbox^4+^ revealing the bowl inversion of nascent and ethylcorannulene.^75^ Unfortunately, despite all efforts, tested solvent systems either froze or were too competitive (see ESI Fig. S24†).

### Incorporation of CPYs into lipid bilayer membranes

**Fig. 7:**
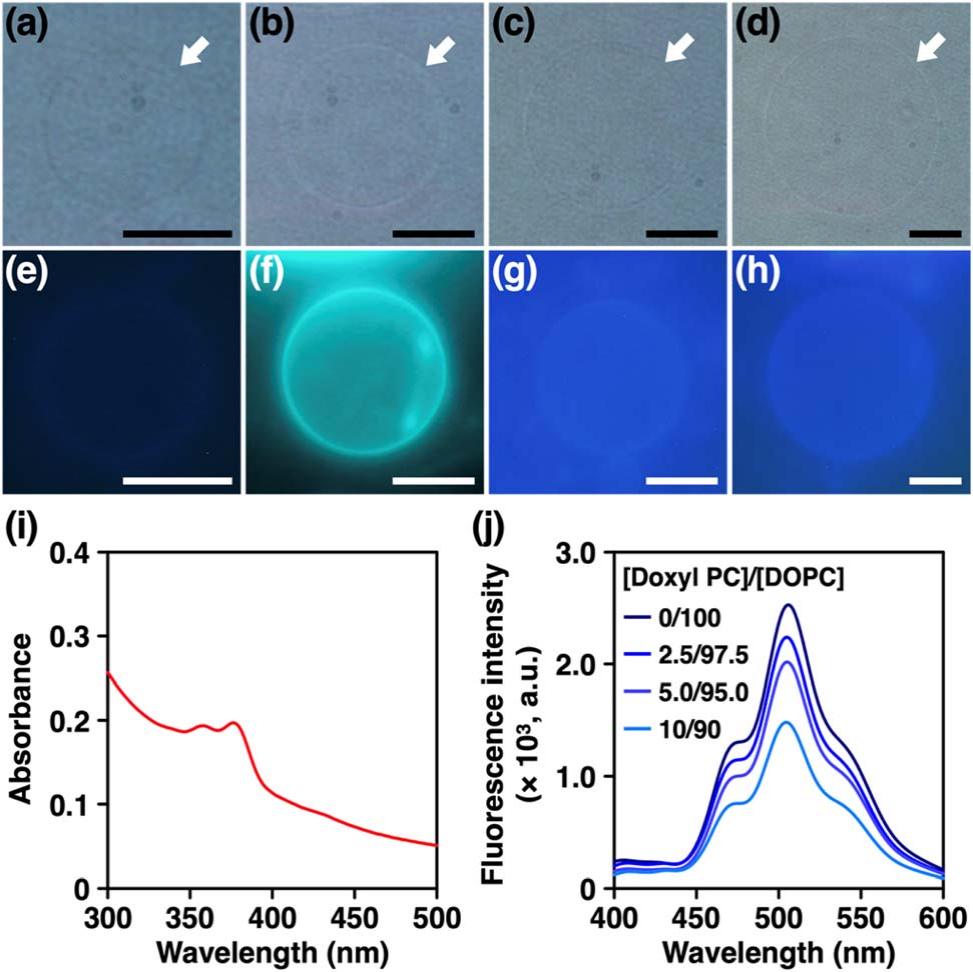
(a–d) Phase contrast and (e–h) fluorescence micrographs (*λ*_ex_ = 330–385 nm, *λ*_obsd_ > 420 nm) of DOPC GUVs ([DOPC] = 200 µM) in aqueous glucose ([glucose] = 200 µM) in the presence of (a and e) 1_[4]_, (b and f) 1_[5]_, (c and g) 1_[6]_, and (d and h) 1_[7]_ ([1_[n]_] = 1 µM) at 25 °C. Scale bars: 10 µm. (i) Absorption spectrum of 1_[5]_-containing DOPC LUV ([1_[5]_] = 5 µM, [DOPC] = 1 mM) in HEPES buffer ([HEPES] = 20 mM, [NaCl] = 50 mM, pH 7.1) at 25 °C. (j) Emission spectra (*λ*_ex_ = 375 nm) of 1_[5]_-containing DOPC LUV with variable content of doxyl PC ([1_[5]_] = 1 µM, [total PC] = 200 µM in HEPES buffer ([HEPES] = 20 mM, [NaCl] = 50 mM, pH 7.1) at 25 °C. [doxyl PC]/[DOPC] = 0/100 (dark blue), 2.5/97.5 (blue), 5.0/95.0 (purple), 10/90 (light blue).

Previously, nanocarbon materials such as carbon nanotubes and fullerenes have been incorporated into the hydrophobic region of lipid bilayer membranes and their physicochemical properties have been studied.^30,35,76–81^ Here, inspired by the slipped stacked tubular stacking of 1_[4-6]_ in the crystalline solid state (see ESI Fig. S13†) reported previously,^52^ we also aimed to explore the ability of 1_[_*_n_*_]_ to be incorporated into membranes. First, we prepared giant unilamellar vesicles (GUVs) formed from 1,2-dioleoyl-*sn*-glycero-3-phosphocholine (DOPC) by a gentle hydration method in the presence of 1_[_*_n_*_]_ (see ESI Section S7.1†). As shown in Fig. 7a–d, phase-contrast microscopy visualised the formation of micrometre-sized GUVs regardless of the ring size of nanohoops. We then examined the corresponding fluorescence micrographs and found that the GUVs prepared in the presence of 1_[5]_ showed strong emission along their edges, while GUVs with other nanohoops did not (Fig. 7e–h). These results indicate that 1_[5]_ was most preferentially localised at the membranes. ^35,36,55^ We have also prepared GUVs with different lipid headgroups and investigated their capability to incorporate 1_[5]_. As shown in Fig. S26,† fluorescence microscopy of GUVs containing phosphatidylcholines (PC), phosphatidylethanolamines (PE), and phosphatidylserines (PS) in the presence of 1_[5]_ exhibited similarly clear emission along the edge of the GUVs, demonstrating that 1_[5]_ can be incorporated into the membranes regardless of the type of lipid headgroups. To further elucidate the localisation of 1_[5]_, we prepared large unilamellar vesicles (LUVs) by extrusion method (see ESI Section S7.2†) and applied spectroscopic techniques. The electronic absorption spectrum of LUVs prepared with 1_[5]_ showed absorption bands at 350–400 nm, characteristic of 1_[5]_ (Fig. 7i).^52^ We then prepared LUVs from DOPC and variable amounts of 1-palmitoyl-2-stearoyl-(12-doxyl)-*sn*-glycero-3-phosphocholine (doxyl PC), which carries a spin-labelled doxyl group in its alkyl tail (see ESI Section S7.3†). The doxyl PC is known to quench the fluorescence of nearby chromophores and can thus be used as a probe to determine the location of chromophores in the membrane. ^35,36,55^ As shown in Fig. 7j, the fluorescence intensity of 1_[5]_ decreased monotonically with increasing doxyl-PC content. These results strongly suggest that 1_[5]_ is localised in the hydrophobic region of the lipid bilayer membranes, similar to other nanocarbon materials.^30,35,76–81^

To explore the functions of 1_[5]_, we tested its potential to transport ions across lipid bilayer membranes, expecting its pore to function as a transmembrane channel or ionophore. For this purpose, we prepared LUVs encapsulating 8-hydroxypyrene-1,3,6-trisulfonic acid (HPTS), a pH-sensitive fluorescent dye (see ESI Section S7.4†). We incorporated 1_[5]_ and then created a pH gradient across the membranes so that the transmembrane ion transport could be monitored by an increase in the fluorescence intensity of HPTS. ^35,36,55^ However, as shown in Fig. S27,† we did not observe any changes in fluorescence intensity. We also performed current recording measurements of 1_[5]_-containing planar lipid bilayer membranes under the application of voltage, but we did not observe any current signals (Fig. S28†). These results suggest that 1_[5]_ does not possess transmembrane ion transport properties.

### Molecular dynamics simulations of 1_[5]_ in lipid bilayer membranes

**Fig. 8:**
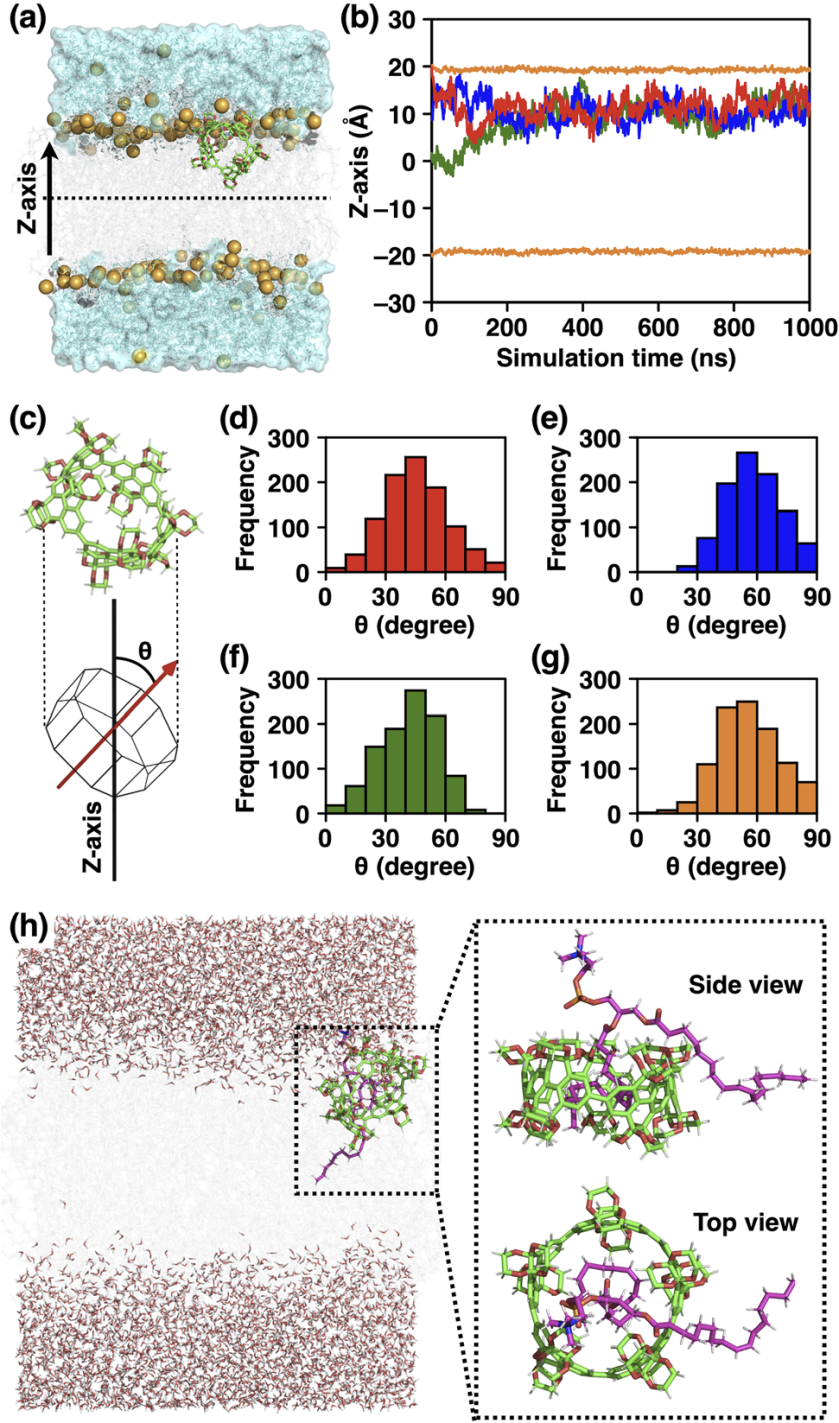
(a) Snapshot at 1 µs of 1_[5]_ in the membrane-water system from MD simulation starting from *z* = 0. The *z*-axis was set to be perpendicular to the membrane plane. 1_[5]_ and lipid molecules are shown in a stick model and phosphorus atoms are shown as orange spheres. (b) The time evolution of the *z*-coordinate of the center of gravity of 1_[5]_ starting from *z* = 0 Å (green), 10 Å (blue), and 20 Å (red). The average coordinates of the phosphorous atoms of the upper and lower leaflets of the bilayers are shown in orange. (c) Structure model of 1_[5]_. The angle between the central axis and the *z*-axis was defined as *Θ*. (d–g) Histograms of the angles *Θ* collected at every 1 ns during each 1 µs MD simulations starting from (d) *z* = 0 Å and *Θ* = 0°, (e) *z* = 0 Å and *Θ* = 90°, (f) *z* = 10 Å and *Θ* = 0°, and (g) *z* = 10 Å and *Θ* = 90°. (h) Various representative snapshots at 1 µs of 1_[5]_ incorporating the alkyl tail of DOPC within its cavity, starting from *z* = 0 Å and *Θ* = 0°.

To further understand the molecular events in the membranes at the atomic level, we performed molecular dynamics (MD) simulations. We first investigated the position of 1_[5]_ within the membranes by running the 1 µs simulations starting from three different initial positions (see ESI Fig. S27a, c and e†). As shown in Fig. 8a, the centre of gravity of 1_[5]_ moved toward the region 10 Å from the hydrophobic bilayer centre within the first 200 ns, regardless of the initial position of 1_[5]_ (Fig. 8b). These results are also consistent with the fluorescence quenching experiments mentioned above (Fig. 7j). Next, we investigated the orientation of 1_[5]_ within the membranes. We assumed that 1_[5]_ adopts a polygonal prismatic structure and parameterised the orientation of 1_[5]_ by defining the angle between the central axis and the *z*-axis as *Θ* (Fig. 8c). We also ran the 1 µs simulation starting from four different initial configurations of 1_[5]_ (see ESI Fig. S27a–d†). As shown in Fig. 8d–g, the most frequent *Θ* was observed between 40 and 50°, regardless of the initial configurations.

To understand the reason for the biased *Θ* value, we analysed the trajectories during the simulations and unexpectedly found that the alkyl tail of DOPC molecule was incorporated into the cavity of 1_[5]_ (Fig. 8h). Similar to the host–guest complexes of aromatic molecular nanocapsules and hydrocarbons reported by Rebek and coworkers,^82,83^ the flexible alkyl tail of DOPC adopted a folded conformation to fill the inner void of 1_[5]_. The biased *Θ*-value of 1_[5]_ can be understood as the result of this complexation event. The complexation also explains the reason for the inability of 1_[5]_ to transport ions across the membranes, because the inner cavity of 1_[5]_ is fully occupied. This complexation capability of 1_[5]_, along with the enhanced solubility of smaller ring-sized CPYs (*n* = 4–6), are likely the main contributing factors that drive the size-preferential incorporation of 1_[5]_ into the membrane.

Experimental efforts were then undertaken to investigate the binding ability of 1_[5]_ with DOPC (up to 10 equiv.). Unfortunately, after ^1^H NMR isothermal titrations of 1_[5]_ with DOPC in benzene-*d*_6_ at 298 K, no binding was observed (see ESI Fig. S25†). The association constant of the weak CH–π interactions between DOPC alkyl tail and 1_[5]_ are likely below the detection limit, with the hydrophobic environment of the bilayer membrane likely driving the complexation event to occur. Replicating this environment for ^1^H NMR titrations remained unachievable as CPYs are insoluble in aqueous media.

These results demonstrate the intriguing function of nanohoops interacting with phospholipids, one of the vital components of life,^84^ and their potential ability to act as supramolecular sensors or functional modulators of cellular membranes.

## Conclusion

An alternative synthetic route towards CPYs was developed which implements a stannylation-based precursor, producing purer material than the previous borylation approach, enabling the growth of single-crystals of the Pt-macrocycle. Reductive elimination of these single-crystals achieved significantly higher selectivity and yields towards smaller ring-sized nanohoops (*n* = 4–6). Encapsulation of polycyclic (aromatic)hydrocarbons within the five-fold CPY, 1_[5]_, were conducted. These studies illustrate the importance of molecular shapes and CH–π contacts—with 1_[5]_ binding with both corannulene (9) (990 M^−1^) and pyrene (10) (195 M^−1^). In addition, 1_[4-7]_ were incorporated into the hydrophobic layer of lipid bilayer membranes. Using spectroscopic techniques and MD simulations, we revealed these molecular events in the membranes at the atomic scale, and unexpectedly discovered the possibility of 1_[5]_ forming a complex with an alkyl tail of the phospholipid. Hence, we hypothesise that incorporation of 1_[5]_ into a lipid bilayer membrane lacking the guest-competitive alkyl chains, which possibly also interfere with the formation of tubular packing structures of 1_[5]_, may allow the formation of channels and host–guest binding required for artificial transport of molecular cargo. We expect that our findings contribute towards the design of functional supramolecular materials as bioimaging tools, capable of interacting with biomolecules, or forming supramolecular assemblies, when integrated within life-inspired artificial systems and living organisms.

## Author contributions

K. C. and N. G. contributed equally and have the right to list their name first in their CV or any bibliographic list. The manuscript was written through contributions of all authors. All authors have given approval to the final version of the manuscript. The authors declare no competing financial interest.

## Conflicts of interest

There are no conflicts to declare.

## Supplementary Material

SC-015-D4SC03408B-s001

SC-015-D4SC03408B-s002

## Data Availability

The data supporting this article have been included as part of the ESI.†
