# Supplementary material for: Nanohoops in membranes: confined supramolecular spaces within phospholipid bilayer membranes
Source: Chem Sci. 2024 Sep 11;15(39):16367–76. doi: 10.1039/d4sc03408b (PMC11409858; doi:10.1039/d4sc03408b)
Supplement: SC-015-D4SC03408B-s001 [file SC-015-D4SC03408B-s001.pdf]

Supplementary Information (SI) for Chemical Science.
This journal is © The Royal Society of Chemistry 2024

Electronic Supplementary Information for

Nanohoops in Membranes: Confined Supramolecular Spaces within Phospholipid Bilayer Membranes

Kylie Chinner,[§,a] Niklas Grabicki,[§,a] Rei Hamaguchi,[b] Mitsunori Ikeguchi,[c] Kazushi Kinbara,[b,d] Sayaka Toyoda,[e] Kohei Sato,[b,e,] and Oliver Dumele[a,f*]*

[a] Department of Chemistry, Humboldt Universität zu Berlin, Brook-Taylor-Str. 2, 12489 Berlin, Germany; [b] School of Life Science and Technology, Tokyo Institute of Technology, Yokohama, Kanagawa 226-8501, Japan.; [c] Graduate School of Medical Life Science, Yokohama City University, Yokohama, Kanagawa 230-0045, Japan.; [d] Research Center for Autonomous Systems Materialogy (ASMat), Institute of Innovative Research, Tokyo Institute of Technology, Kanagawa 226-8501, Japan.; [e] Department of Chemistry, School of Science, Kwansei Gakuin University, Sanda, Hyogo 669-1330, Japan. orcid.org/0000-0002-8948-8537; Email: ksato@kwansei.ac.jp; [f] Institute of Organic Chemistry, University of Freiburg, 79104, Freiburg, Germany; E-mail: oliver.dumele@oc.uni-freiburg.de. Homepage: www.dumelelab.com

Table of Contents

S1. General Information....2
S2. Synthetic Procedures....5
S3. Selected NMR Spectra....11
S4. Single-crystal X-ray Data....16
S5. Geometrical Considerations and Volume Analysis of the Host $\mathbf{1}_{[5]}$....21
S6. Host-guest Binding Experiments by ^{1}H NMR Titration with $\mathbf{1}_{[5]}$....23
S7. Preparation of vesicles....33
S8. MD simulations....37
S9. References....39

S1. General Information

Reagents (Acros, AlfaAesar, Sigma-Aldrich, ABCR, Fluorochem, and TCI) were purchased as reagent grade and used without further purification, unless otherwise specified.

Solvents for synthesis were dried using a Pure Solv Micro Solvent Purification System from Innovative Technology and stored over molecular sieves 3–4 Å.

Glassware. All non-aqueous reactions were performed in oven-dried glassware and under a N_2 atmosphere.

Automated Medium Pressure Column Chromatography (MPLC) was performed on a Teledyne ISCO CombiFlash R_f 300 system with 200 mL/min max flow, 200 psi, equipped with integrated ELSD and 200–800 nm UV/Vis variable wavelength detector.

High-Performance Liquid Chromatography (HPLC) was run on a Waters 600 HPLC System equipped with a Waters 600 solvent pump, a Waters 600 controller, and a Waters 996 photodiode array detector.

Ultra-Performance Liquid Chromatography coupled with Mass Spectrometry (HPLC/MS) was performed with a Waters ACQUITY HPLC H-Class, equipped with a quaternary solvent manager (QSM), a sample manager-flow through needle (SM-FTN), a column heater, a column manager, an ACQUITY HPLC BEH Phenyl 1.7 µm, 2.1 × 100 mm column, an ACQUITY HPLC BEH C18 1.7 µm, 2.1 × 100 mm column and a photodiode array detector (PDA eλ), and an ACQUITY QDa detector. The mobile phase is a gradient of MeCN/H_2O individually optimised for each separation.

Recycling gel permeation chromatography (GPC) was performed with a JAI LC-9210NEXT using trichloromethane with 4% ethanol as a stabiliser as the eluent. The GPC was equipped with the following set of columns: PSS SDV 50 Å, 20 × 600 mm; PSS SDV 1000 Å, 20 × 600 mm; Jaigel-2H 5 Å, 20 × 600 mm. The flow rate was kept at 4.5 mL min^{-1}.

Thin layer chromatography (TLC) was conducted on aluminum sheets coated with SiO_2-60 F_{254} obtained from Merck; visualisation with a UV lamp (254 or 366 nm).

Evaporation *in vacuo* was performed at 40–60 °C and 700–10 mbar. All products were dried under vacuum (ca. 10^{-2} mbar) before analytical characterisation.

Reported yields refer to spectroscopically and chromatographically pure compounds that were dried under high vacuum (ca. 10^{-2} mbar) before analytical characterisation, unless otherwise specified.

Nuclear magnetic resonance (NMR) spectra were recorded using a Bruker Avance II 300 (300 MHz for ^{1}H and 75 MHz for ^{13}C) and a Bruker Avance II 500 (500 MHz for ^{1}H and 126 MHz for ^{13}C) at room temperature and are reported as follows: chemical shift (d) in ppm (multiplicity, coupling constant *J* in Hz, number of protons; assignment). The residual deuterated solvent was used as the internal reference ($CDCl_3$: δ_H = 7.26 ppm, CD_2Cl_2: δ_H = 5.32 ppm, CD_3OD: δ_H = 3.31 ppm, $(CD_3)_2SO$: δ_H = 2.50 ppm; ($CDCl_3$: δ_C = 77.16 ppm, CD_2Cl_2: δ_C = 54.00 ppm, CD_3OD: δ_C = 49.00 ppm, $(CD_3)_2SO$: δ_C = 39.52 ppm; The resonance multiplicity is described as s (singlet), d (doublet), t (triplet), q (quartet), quint (quintet), sept. (septet), m (multiplet), and br. (broad).

Melting Points (m.p.) were measured using an MPM-H_2 by Schorpp Gerätetechnik in open capillaries and are uncorrected.

Infrared (IR) spectra were recorded on a Vertex 70v spectrometer by Bruker equipped with a diamond ATR attachment and are basegraph-corrected. The spectra were measured between 4000 and 400 cm^{-1}. Absorption bands are reported in wavenumbers (cm^{-1}) and their relative intensities described as s (strong), m (medium), or w (weak).

High-resolution mass spectrometry (HRMS) data were recorded on a Q-Exactive by Thermo Fisher via direct injection at flow rates between 20 and 100 µL min^{-1} in positive and negative mode, a resolution of 35000, an AGC Target Value of 106, a maximum inject time of 50 ms, and mass ranges between 200 and 1000 *m*/*z*.

Mass spectrometry (MS) data were recorded on a MALDI-TOF Autoflex Max (Bruker Daltonik, Bremen), 355 nm laser, 4000 shot accumulated on 2 different spots, reflector modus, Matrix DCTB (*trans*-2-[3-(4-*tert*-butylphenyl)-2-methyl-2-propenylidene]) 20 mg mL^{-1} in $CHCl_3$ + 1 mg K trifluoroacetate 3 50 µL matrix solution pre-mixed with 20 µL sample solution (approximately 0.1 mg mL^{-1} in $CHCl_3$) 1 µL droplet deposited of premixed solution on stainless steel target recording using FlexControl software (Bruker).

Single crystal X-ray data were measured with a BRUKER D8 VENTURE area detector with Mo-Kα radiation (λ = 0.71073 Å). Multi-scan absorption corrections implemented in SADABS[1] were applied to the data. The structures were solved by intrinsic phasing method

(SHELXT-2013)[2] and refined by full matrix least square procedures based on F2 with all measured reflections (SHELXL-2014)[3] in the graphical user interface (OLEX2)[4] with anisotropic temperature factors for all non-hydrogen atoms. All hydrogen atoms were added geometrically and refined by using a riding model. The calculation of the voids resulting from heavily disordered solvent molecules in the structure was achieved using the tool implemented in OLEX2[5] or by using the PLATON/SQUEEZE protocol.[6],[7]

Phase contrast and fluorescence microscopy for Fig. 7 were performed with an Olympus IX-71 microscope equipped with a U-MWU2 mirror unit (excitation filter: λ = 330–385 nm, emission filter: λ = 420 nm, dichroic mirror: λ = 400 nm). Phase-contrast and fluorescence microscopy for ESI Figure S26 were performed on an Olympus model IX73 inverted microscope, using a U-FUW mirror unit (excitation filter: λ = 340–390 nm, emission filter: λ = 420 nm, dichroic mirror: λ = 410 nm) for fluorescence imaging.

Electronic absorption spectra were recorded on a JASCO model V-650 UV-vis spectrophotometer using a quartz cell of 10 mm optical path length.

Fluorescence spectra were recorded on a JASCO model FP-6500 spectrofluorometer using a quartz cell of 10 mm optical path length.

Current recording measurement was performed on a Nanion Technologies model Orbit mini platform.

S2. Synthetic Procedures

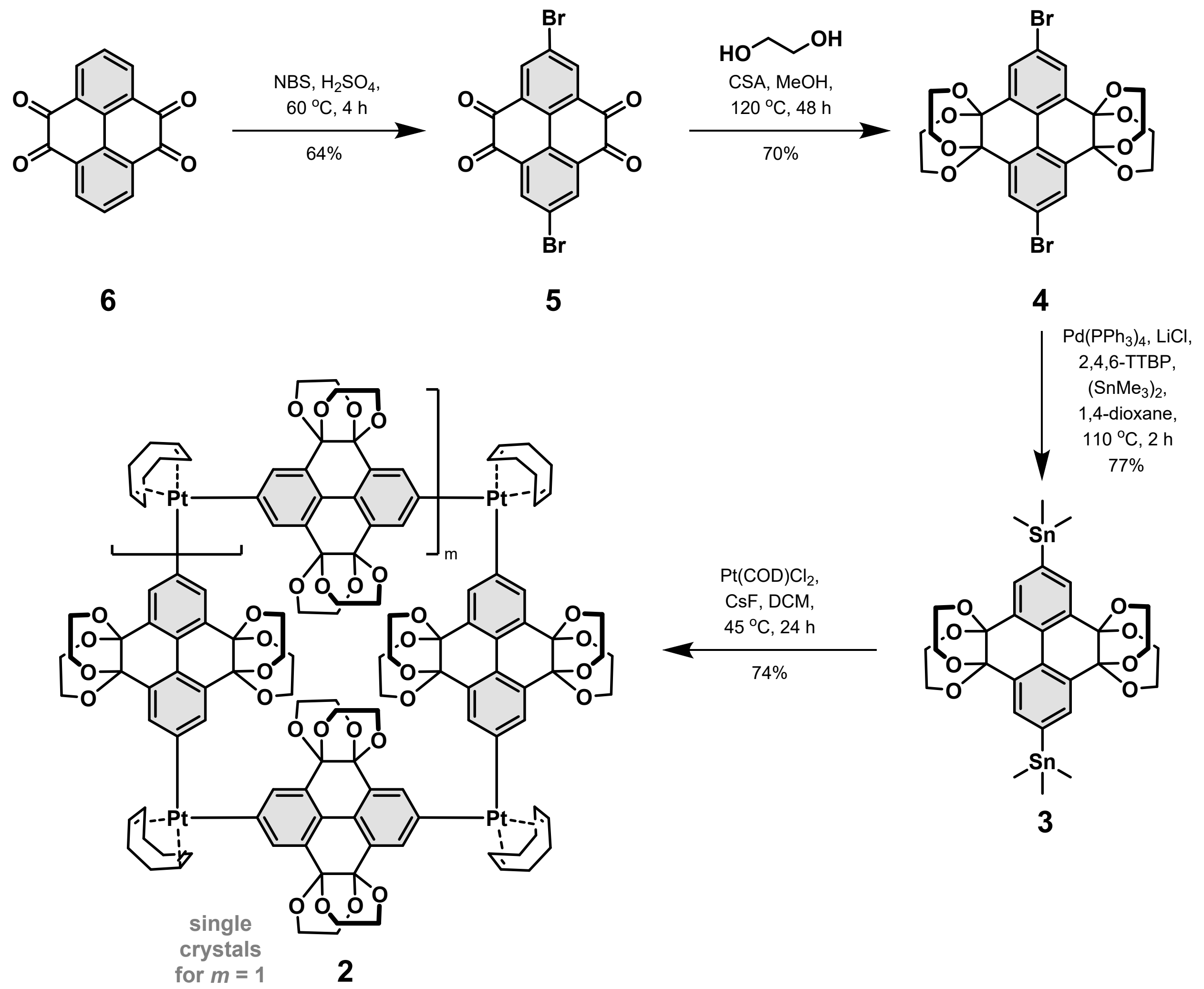

Scheme S1 Overview on the alternative synthetic approach to access Pt-macrocycle **2**.

2,7-Bisbromo-pyrene-4,5,9,10-tetrone (5)[8]

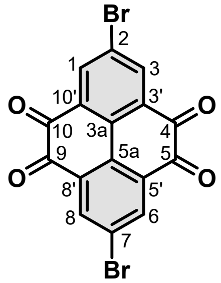

The following procedure was adapted from the literature and modified.[8] A solution of pyrene-4,5,9,10-tetrone[9] (**6**) (5.6 g, 21.3 mmol) was dissolved in concentrated H_2SO_4 (95–97%, 270 mL). The reaction mixture was degassed with a constant flow of N_2. NBS (11.4 g, 63.8 mmol) was added gradually over 4 h at 25 °C. The reaction mixture was stirred for 18 h, poured over ice, filtered and suspended in MeOH. The MeOH suspension was filtered, and the resulting yellow solid washed several times with MeOH. Drying under high vacuum afforded **5** (7.8 g, 87%) as a yellow solid. The characterisation data is in agreement with the literature.[8] R_f = 0.15 (SiO_2; cyclohexane/EtOAc 1:2); ^{1}H NMR (500 MHz, DMSO-d_6, 25 °C): δ = 8.36 ppm (s 4H, 4 H–C(1,3,6,8)); ^{13}C NMR did not resolve in sufficient signal to noise ratios in common organic NMR solvents; HR-ESI-MS: *m/z*: 419.8450 ([M + H]$^+$ calcd. for $C_{16}H_7O_4^+$: 419.8456).

2,7-Dibromo-pyrene-4,5,9,10-tetra(ethyleneglycol)ketal (4)[10]

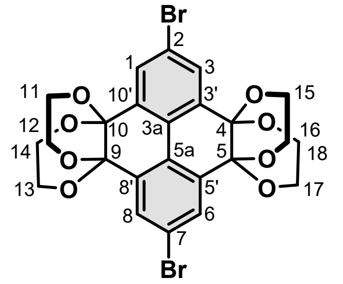

Based on a modified procedure in the literature,[10] a suspension of dibromide **5** (4.1 g, 9.8 mmol), ethylene glycol (11.0 mL, 0.20 mol), (+)-camphorsulfonic acid (0.34 g, 1.5 mmol), in dry THF (28 mL) was degassed with a constant flow of N_2 in an oven-dried 100 mL-pressure tube, sealed, and heated to 120 °C for 24 h. The mixture was cooled to 23 °C, filtered and subsequently was washed with MeOH (3 x 60 mL) and diethyl ether (3 x 50 mL), and dried under high-vacuum to obtain **4** (3.9 g, 68%) as a beige solid. The characterisation data agrees with the literature.[10]

R_f = 0.1 (SiO_2; cyclohexane/EtOAc 1:2); ^{1}H NMR (500 MHz, CD_2Cl_2, 25 °C): δ = 7.88 (s, 4H, 4 H–C(1,3,6,8)), 4.18 (br., 8H, 8 H–C(11–18)), 3.64 ppm (br., 8H, 8 H–C(11–18)); ^{13}C NMR (126 MHz, CD_2Cl_2, 25 °C, assignments based on ^{1}H,^{13}C-HSQC NMR spectra): δ = 135.68 (C(2,7)), 131.03 (C(1,3,6,8)), 127.67 (C(3a,5a)), 124.29 (C(3',5',8',10')), 92.44 ppm (C(4,5,9,10)), the signal for the carbon atoms of the ethylene glycol groups are not observed, due to the dynamics of six-membered rings; HR-ESI-MS: *m/z*: 596.9577 ([M + H]$^+$ calcd. for $C_{24}H_{21}Br_2O_8^+$: 596.9583).

2,7-Bis(trimethylstannyl)-pyrene-4,5,9,10-tetra(ethyleneglycol)ketal (3)[10]

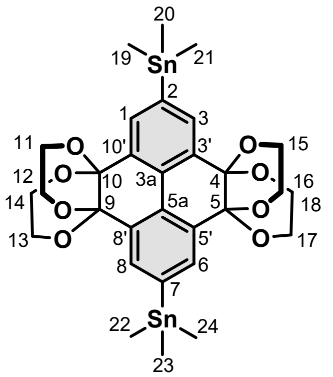

Dibromide 9 (1.5 g, 2.5 mmol), 2,4,6-tri-tert-butylphenol (0.7 mg, 0.25 mmol), lithium chloride (0.5 g, 12.6 mmol) were suspended in dry 1,4-dioxane (90 mL) and degassed with a constant flow of N_2 in an oven-dried 350 mL-pressure tube. 1,1,1,2,2,2-hexamethyldistannane (2.06 g, 6.3 mmol) and tetrakis(triphenylphosphine)-palladium(0) (0.29 g, 0.25 mmol) were added afterwards and the reaction mixture was sealed and heated to 110 °C for 2 h. The reaction mixture was allowed to cool down to 25 °C and filtered over a pad of silica. The solvent was evaporated under reduced pressure (caution: must be performed in a fume hood; all instruments should be cleaned thoroughly afterwards due to remaining organostannanes). Purification by preparative MPLC (SiO_2, gradient cyclohexane/CH_2Cl_2 3:1→0:1) and drying under vacuum gave **3** (1.48 g, 77%) as a colourless solid.

R_f = 0.4 (SiO_2; CH_2Cl_2); ^{1}H NMR (500 MHz, CD_2Cl_2, 25 °C): δ = 7.86 (s, 4H, H–C(1,3,6,8)), 4.19 (br. 8H, H–C(11–18)), 3.64 (br. 8H, H–C(11–18), 0.36 (s, 18H, H–C(19–24)) ppm; ^{13}C NMR (126 MHz, CD_2Cl_2, 25 °C, assignments based on ^{1}H,^{13}C-HSQC NMR spectra): δ = 145.38 (C(2,7)), 134.76 (C(1,3,6,8)), 132.12 (C(3',5',8',10')), 129.40 (C(3a,5a)), 93.33 (C(4,5,9,10)), 61.82 (br., C(11–18), 9.07 (C(19–24)) ppm; HR-ESI-MS: m/z (%): 765.0677 (**3**, $[M + H]^+$, calcd. for $C_{30}H_{38}O_8Sn_2$ H^+: 765.0457)

[*n*]-Cyclo-2,7-(platinum(cyclooctadiene)pyren-4,5,9,10-tetraketal (2)

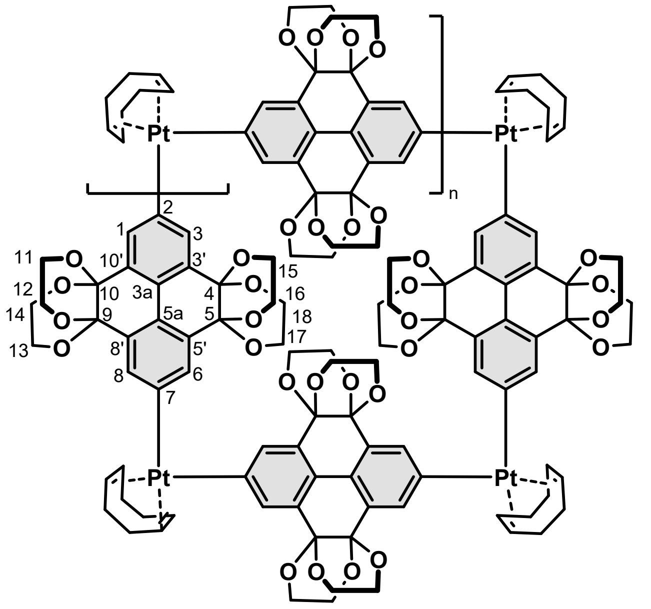

Adapted from the literature,[12] a suspension of dichloro(cycloocta-1,5-diene)platinum(II) (189.7 mg, 507 µmol), CsF (924 mg, 6.1 mmol), and **3** (350.0 mg, 507 µmol) in dry CH_2Cl_2 (140 mL, 5.2 ppm H_2O by Karl-Fischer titration) in an oven-dried 350 mL-pressure tube was degassed using a stream of N_2 for 10 min, sealed, and stirred at 45 °C for 24 h. The mixture was cooled to 23 °C, diluted with cyclohexane (140 mL), and the major volatile CH_2Cl_2 was evaporated to induce precipitation. The resulting suspension was filtered and the pale brown solid was washed with cyclohexane and MeOH. Drying under vacuum afforded **2** (277.5 mg, 74%) as an off-white solid. Colourless crystals of **2** were grown via slow evaporation from a CD_2Cl_2 solution and were used in the next step.

R_f = 0.2 (SiO_2; $CHCl_3$/MeOH 99:1); ^{1}H NMR (400 MHz, CD_2Cl_2, 25 °C): δ = 7.41 (br. s, 16H, 16 H–C(1,3,6,8)), 5.21 (br. s, 16H, 16 H2–C(cod)), 4.35–2.90 (m, 64H, 16 H–C(11–18)), 2.56 ppm (br. s, 32H, 16 H_2–C(cod); ^{13}C NMR measurements gave no sufficient signal-to-noise ratio due to low solubility; (FT)ATR-IR: ν_{max} = 3051 (m), 2949 (m), 2873 (m), 2190 (w), 2045 (w), 1438 (m), 1264 (m), 1176 (m), 1091 (s), 1011 (s), 958 (s), 897 (m), 730 (m), 583 (w) cm^{-1}; MALDI-TOF-MS: *m*/*z* (%): 2995.39 ([*M* + K]$^+$, calcd. for $C_{120}H_{100}O_{40}K^+$: 2995.66).

[*n*]Cyclo-2,7-(4,5,9,10-tetra(ethyleneglycol)ketal)-pyrenylene ($1_{[n=4–6]}$)

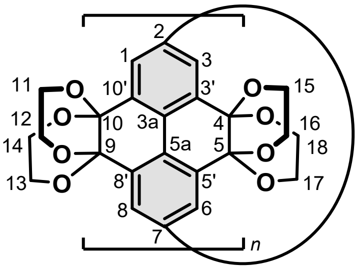

A solution of **2** (50.9 mg, 16.9 µmol) and triphenylphosphine (180.6 mg, 688.5 µmol) in dry 1,2-dichlorobenzene (5 mL) was degassed for 30 min with a constant flow of N_2 under sonication. The reaction vial was sealed and the mixture was heated in a microwave reactor to 180 °C and kept at that temperature for 1 min (typical heating time: 5 min). After cooling to 100 °C, the brown–orange mixture was vacuum-filtered through a PTFE filter (pore size 0.2 µm) to remove insoluble linear oligomers. The filtrate was loaded onto a short pad of silica and was eluted with $CHCl_3$ (ca. 150 mL — major side products and remaining triphenylphosine are first eluted) followed by $CHCl_3$/MeOH 96:4 (ca. 100 mL), which elutes the cyclic product fraction. Evaporation gave a yellow solid (containing macrocyclic products *n* = 4–8 with minor amounts of linear oligomers) that was further purified via *r*GPC (five cycles in sum). Each macrocyclic product fraction (*n* = 4–6) was again purified individually via *r*GPC to obtain analytically pure compounds.

$\mathbf{1}_{[n=4]}$: Yellow solid (6.3 mg, 21% with regard to the total amount of pyrene fragments added to the reaction). R_f = 0.4 (SiO_2; $CHCl_3$/MeOH 96:4); ^{1}H NMR (500 MHz, $CDCl_3$, 25 °C): δ = 7.90 (s, 16H, 16 H–C(1,3,6,8)), 4.23 (br. m, 16H), 4.11 (br. m, 16H), 3.66 (br. m, 16H, 16 H–C), 3.58 ppm (br. m, 16H); ^{13}C NMR (126 MHz, $CDCl_3$, 25 °C, assignments based on ^{1}H,^{13}C-HSQC and HMBC NMR spectra): δ = 139.5 (C(3a,5a)), 134.0 (C(2,7 or 3’,5’,8’10’)), 128.23 (C(1,3,6,8)), 125.7 (C(2,7 or 3’,5’,8’10’)), 93.2 (C(4,5,9,10)) 61.8 (C(11–18)), 61.4 ppm (C(11–18)); MALDI-TOF-MS: *m*/*z*: 1744.42 ([*M*], calcd. for $C_{96}H_{80}O_{32}$: 1744.46).

$\mathbf{1}_{[n=5]}$: Yellow solid (0.9 mg, 3% with regard to the total amount of pyrene fragments added to the reaction). R_f = 0.3 (SiO_2; $CHCl_3$/MeOH 96:4); ^{1}H NMR (500 MHz, $CDCl_3$, 25 °C, assignments based on ^{1}H,^{13}C-HSQC and HMBC NMR spectra): δ = 7.89 (s, 16H, 16 H–C(1,3,6,8)), 4.19 (broad s, 20H, 16 H–C(out of C11–C18)), 4.13 (broad s, 20H, 16 H–C(out of C11–C18)), 3.69 (broad s, 20H, 16 H–C(out of C11–C18)), 3.65 ppm (broad s, 20H, 16 H–C(out of C11–C18)); ^{13}C NMR (126 MHz, $CDCl_3$, 25 °C, assignments based on ^{1}H,^{13}C-HSQC and HMBC NMR spectra): δ = 140.2 (C(3a,5a)), 134.0 (C(2,7 or 3’,5’,8’10’)), 129.1

(C(1,3,6,8)), 125.6 (C(2,7 or 3',5',8'10')), 93.0 (C(4,5,9,10)), 61.7 (C(11–18)), 61.4 ppm (C(11–18)); MALDI-TOF-MS: *m*/*z*: 2180.57 ([*M*], calcd. for $C_{120}H_{100}O_{40}$: 2180.59).

$\mathbf{1}_{[n=6]}$: Yellow solid (0.4 mg, <3% with regard to the total amount of pyrene fragments added to the reaction). R_f = 0.3 (SiO_2; $CHCl_3$/MeOH 96:4); ^{1}H NMR (500 MHz, $CDCl_3$, 25 °C, assignments based on ^{1}H,^{13}C HMBC NMR spectra): δ = 7.89 (s, 24H, 16 H–C(1,3,6,8)), 4.17 (broad s, 48H, 16 H–C(out of C11–C18)), 3.70 (broad s, 24H, 16 H–C(out of C11–C18)), 3.60 ppm (broad s, 24H, 16 H–C(out of C11–C18)); ^{13}C NMR measurements gave no sufficient signal-to-noise ratio due to low solubility; ^{1}H,^{13}C-HMBC NMR spectra, extracted ^{13}C signals (600 MHz, $CDCl_3$, 25 °C) δ = 141.2, 128.8, 125.4, 92.7 ppm; MALDI-TOF-MS: *m*/*z* (%): 2616.76 ([*M*], calcd. for $C_{144}H_{120}O_{48}$: 2616.70).

$\mathbf{1}_{[n=7]}$ and $\mathbf{1}_{[n=8]}$ were not observable for the reductive elimination on single-crystals of **2**.

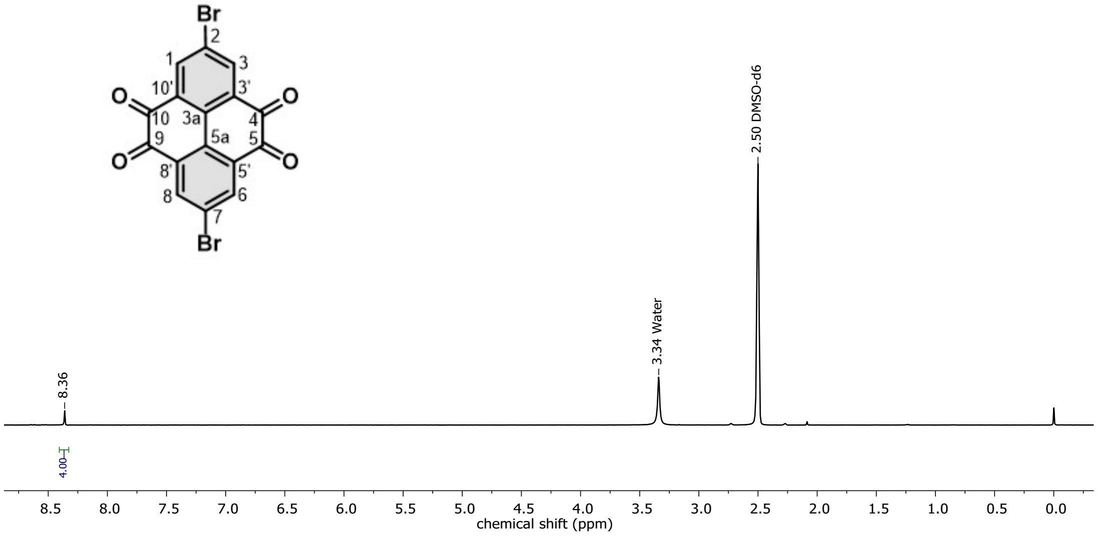

S3. Selected NMR Spectra

Figure S1 ^{1}H NMR spectrum (500 MHz, 298 K) of 2,7-dibromo-pyrene-4,5,9,10-tetrone (**5**) in DMSO-d_6.

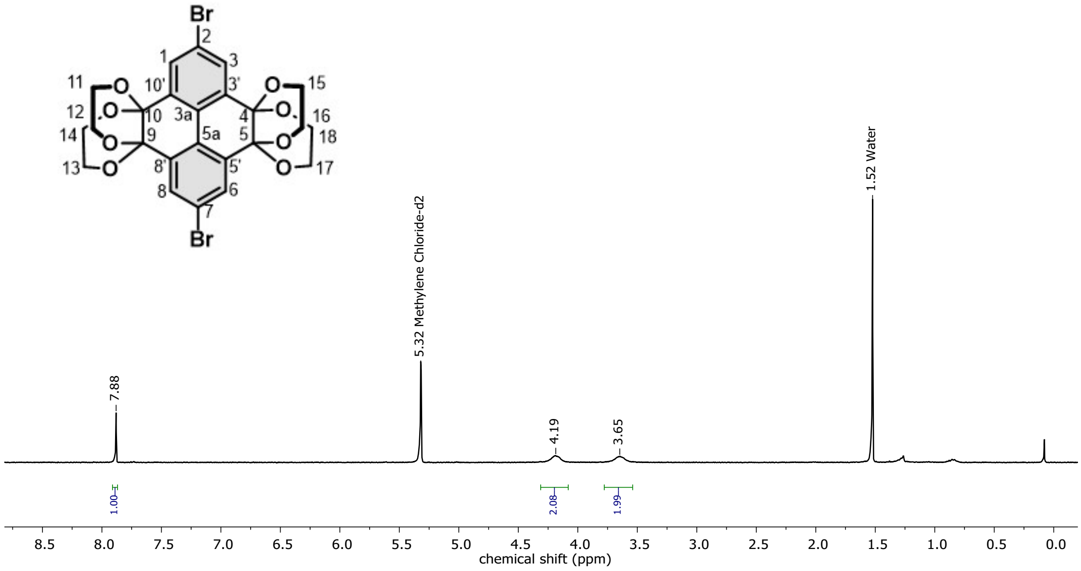

Figure S2 ^{1}H NMR spectrum (500 MHz, 298 K) of 2,7-bisbromo-pyrene-4,5,9,10-tetra(ethyleneglycol)ketal (**4**) in CD_2Cl_2.

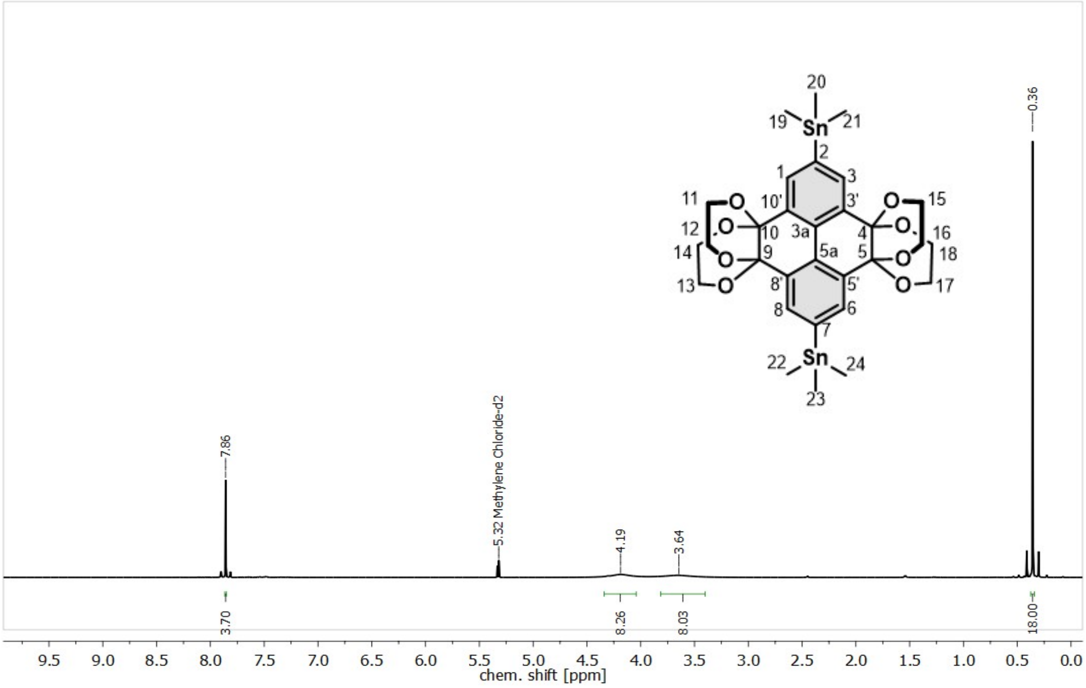

Figure S3 ^{1}H NMR spectrum (500 MHz, 298 K) of 2,7-bis(trimethylstannyl)-4,5,9,10-tetra(ethyleneglycol)ketal-pyrene (**3**) in CD_2Cl_2.

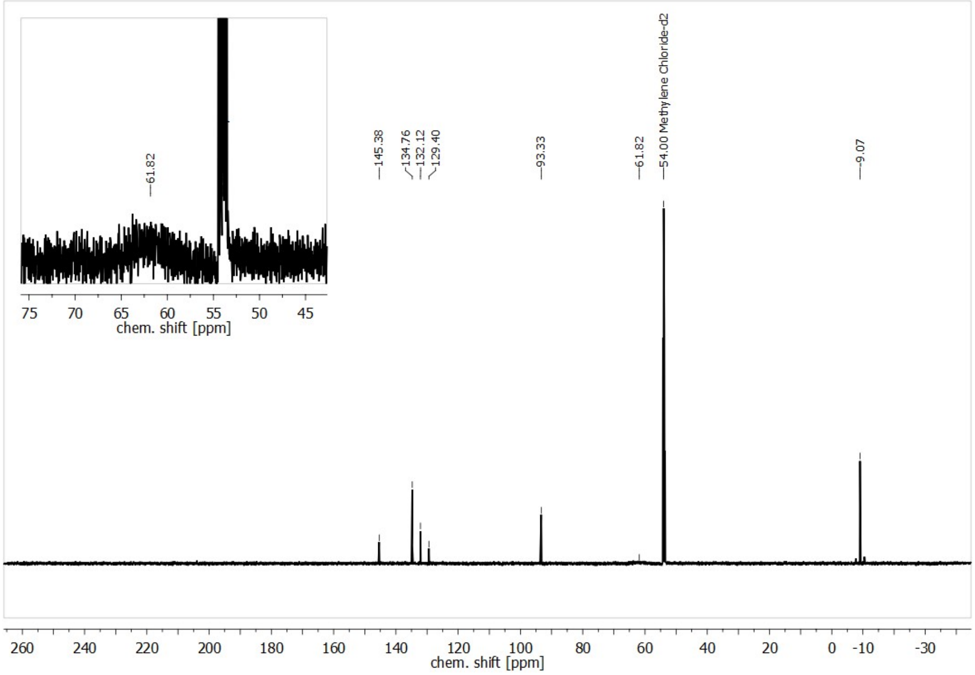

Figure S4 ^{13}C NMR spectrum (126 MHz, 298 K) of 2,7-bis(trimethylstannyl)-4,5,9,10-tetra(ethyleneglycol)ketal-pyrene (**3**) in CD_2Cl_2.

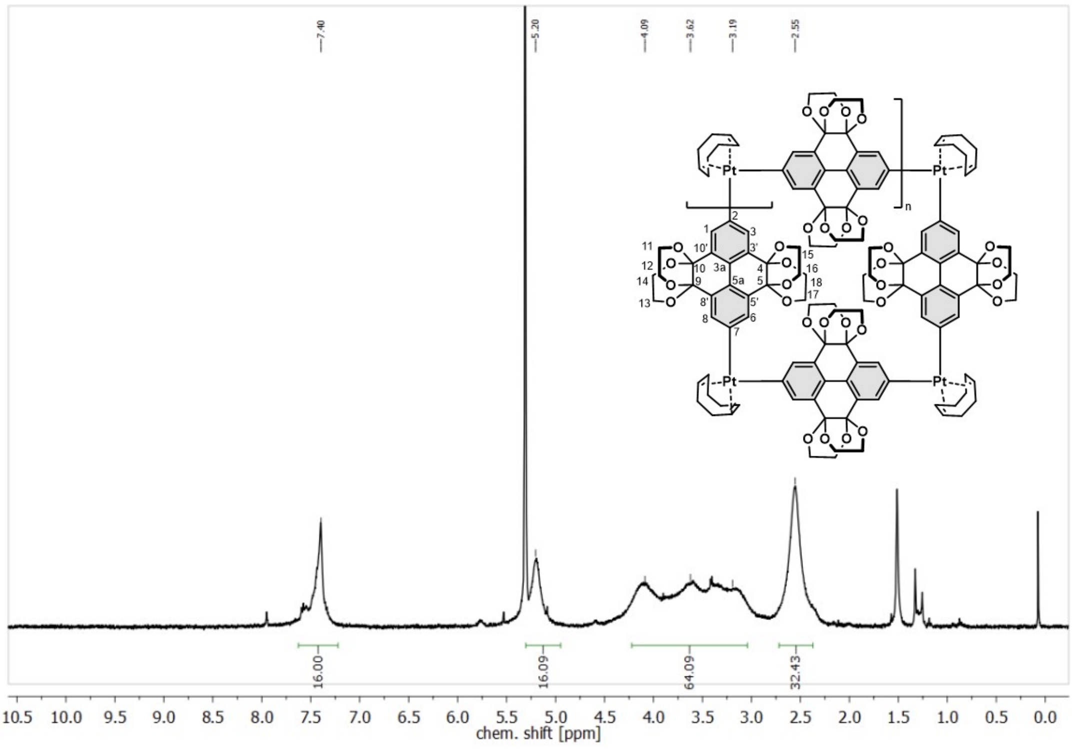

Figure S5 ^{1}H NMR spectrum (500 MHz, 298 K) of [n]-Cyclo-2,7-(platinum(cyclooctadiene)pyren-4,5,9,10-tetraketal (**2**) in CD_2Cl_2.

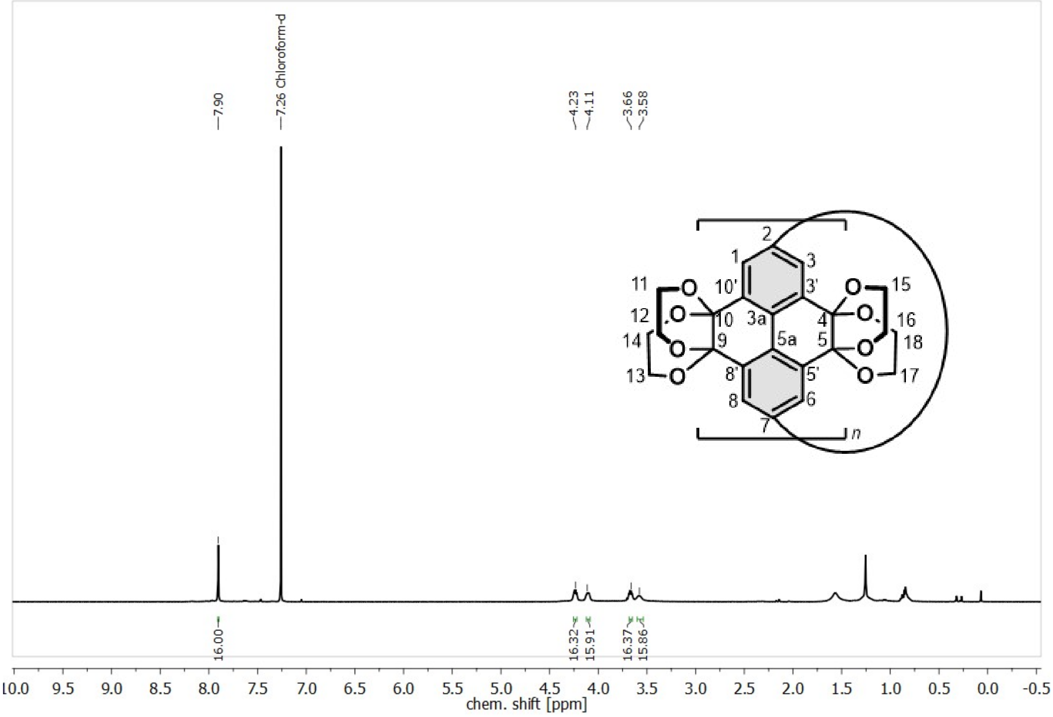

Figure S6 ^{1}H NMR spectrum (500 MHz, 298 K) of [4]cyclo-2,7-pyren-(4,5,9,10-tetra(ethyleneglycol)ketal)ylene ($\mathbf{1}_{[4]}$) in $CDCl_3$.

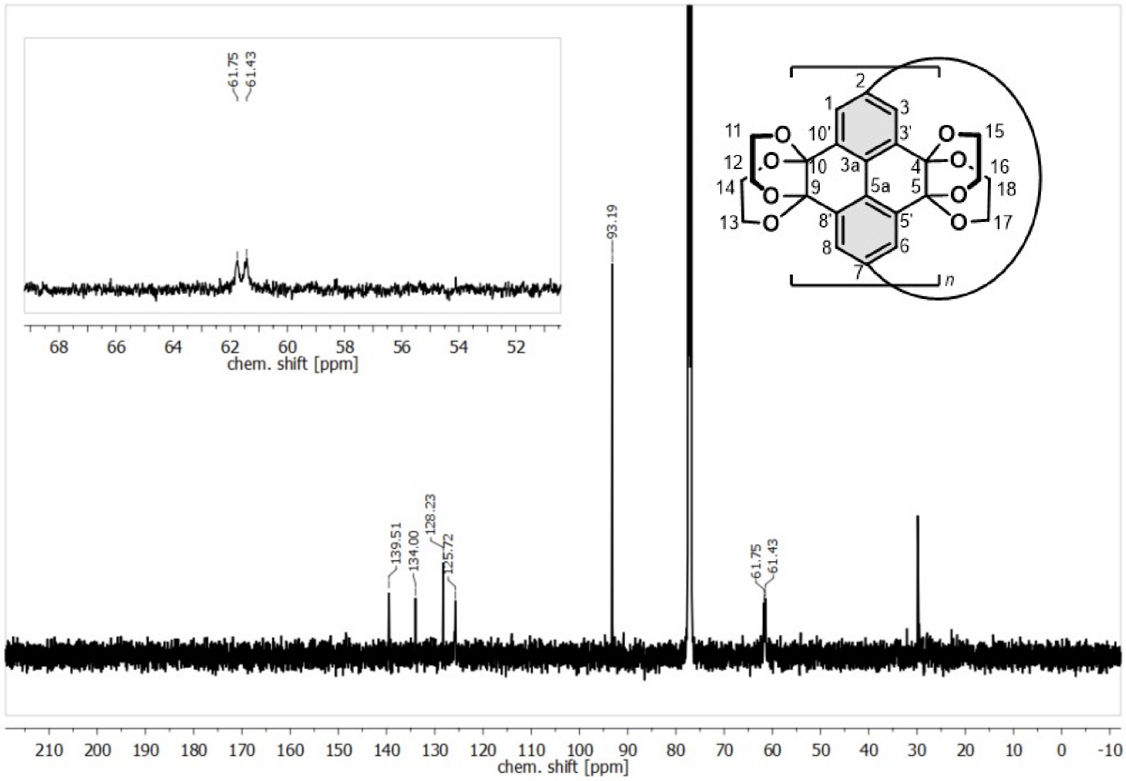

Figure S7 ^{13}C NMR spectrum (126 MHz, 298 K) of [4]cyclo-2,7-pyren-(4,5,9,10-tetra(ethyleneglycol)ketal)ylene ($\mathbf{1}_{[4]}$) in $CDCl_3$.

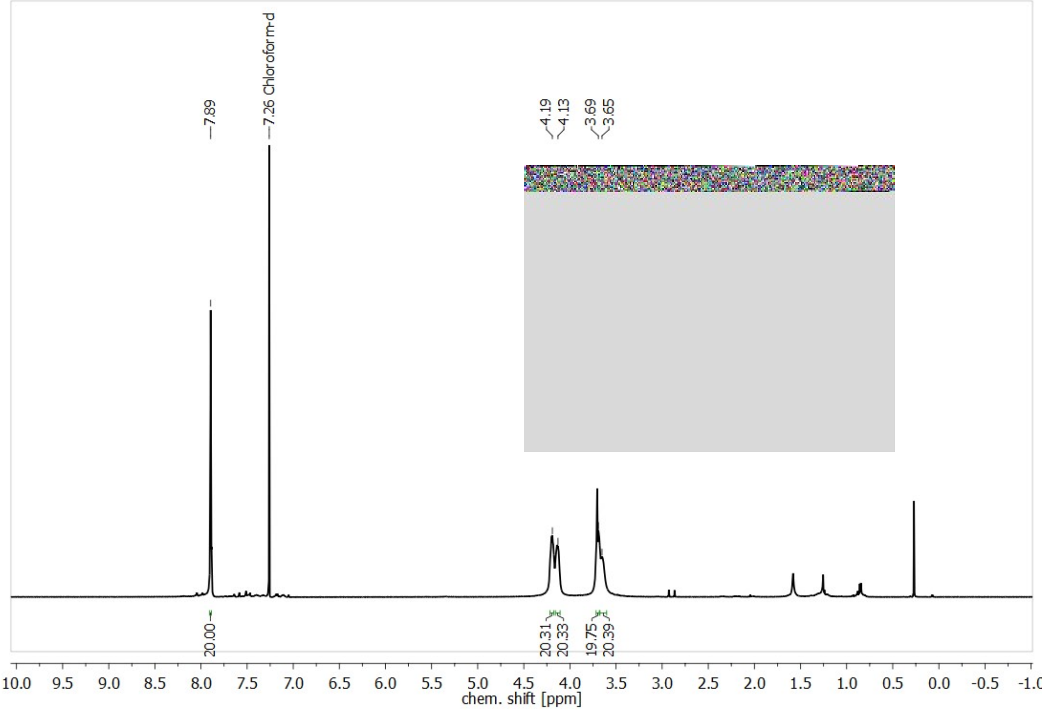

Figure S8 ^{1}H NMR spectrum (500 MHz, 298 K) of [5]cyclo-2,7-pyren-(4,5,9,10-tetra(ethyleneglycol)ketal)ylene ($\mathbf{1}_{[5]}$) in $CDCl_3$.

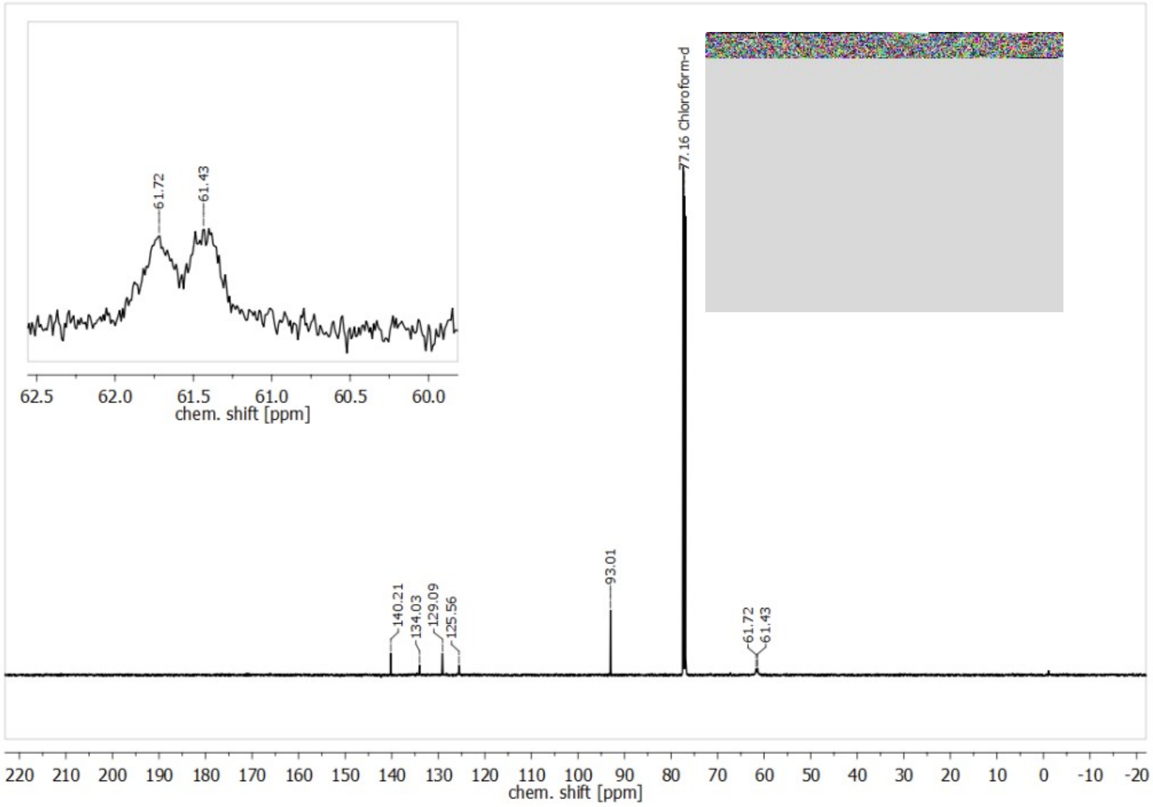

Figure S9 ^{13}C NMR spectrum (126 MHz, 298 K) of [4]cyclo-2,7-pyren-(4,5,9,10-tetra(ethyleneglycol)ketal)ylene ($\mathbf{1}_{[5]}$) in $CDCl_3$.

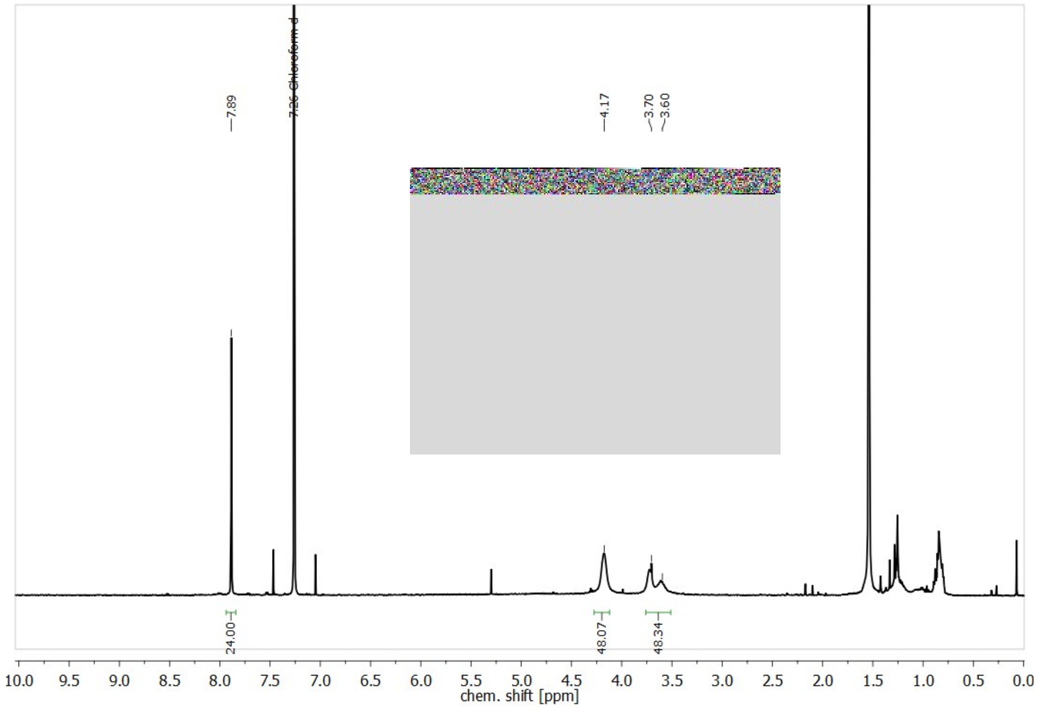

Figure S10 ^{1}H NMR spectrum (500 MHz, 298 K) of [6]cyclo-2,7-pyren-(4,5,9,10-tetra(ethyleneglycol)ketal)ylene (**1**$_{[6]}$) in $CDCl_3$. (solvent residues 5.32 CH_2Cl_2, 1.56 H_2O, and impurities (grease) 0.8 and 1.3 ppm).

S4. Single-crystal X-ray Data

X-ray Crystallography. CCDC-2049559 (**2**$_{[4]}$), CCDC-2236136 (**1**$_{[5]}$···corannulene), contain the supplementary crystallographic data for this paper, including structure factors and refinement instructions. These data can be obtained free of charge from the joint Cambridge Crystallographic Data Centre and Fachinformationszentrum Karlsruhe Access Structures service (Cambridge Crystallographic Data Centre, 12 Union Road, Cambridge CB2 1EZ, UK (fax: +44(1223)-336-033; e-mail: deposit@ccdc.cam.ac.uk), or online via www.ccdc.cam.ac.uk/structures.

S4.1 Single-crystal X-ray Data, Structure Refinement, and Structure Analysis for [*n*]-Cyclo-2,7-(platinum(cyclooctadiene)pyren-4,5,9,10-tetraketal (2$_{[4]}$)

Colourless crystals were grown via slow evaporation from a CD_2Cl_2 solution.

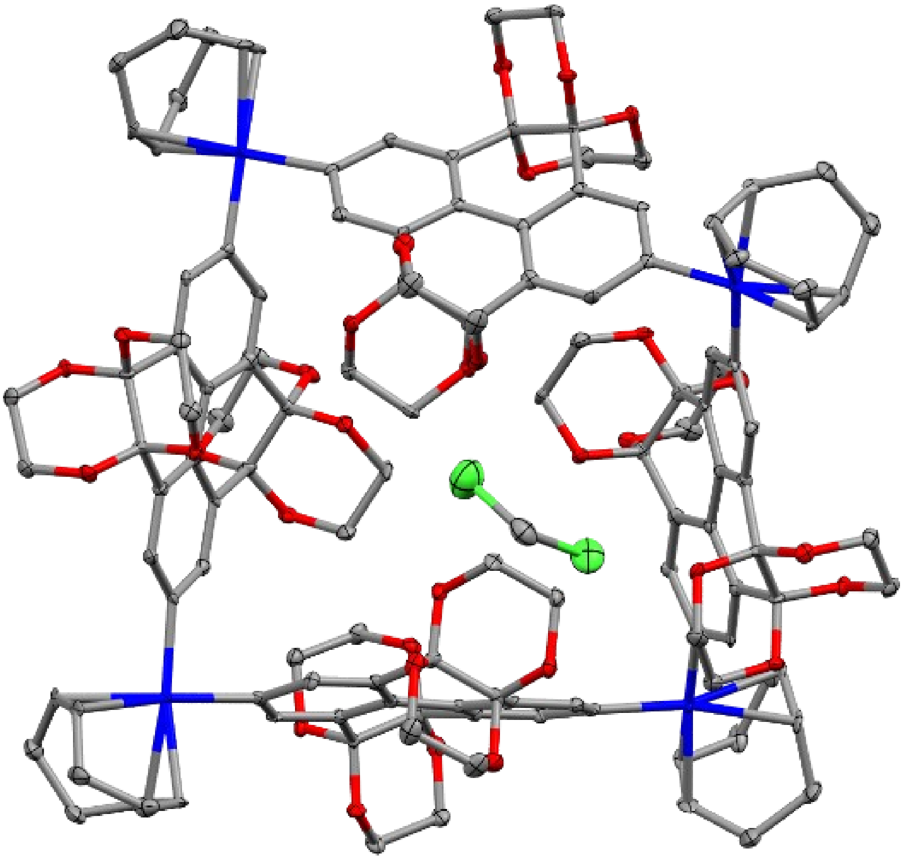

Figure S11 X-ray structure of **2**$_{[4]}$ at 100 K. Ellipsoids are shown at 50% probability, hydrogen atoms and solvent molecules are omitted for clarity. Colour code: carbon, grey; oxygen; red; chlorine, green; platinum, blue.

Table S1 Crystal data and structure refinement for $\mathbf{2}_{[4]}$

CCDC Deposition number	2049559
Empirical formula	$C_{128}H_{128}O_{32}Pt_4 \cdot 4(CH_2Cl_2)$
Formula weight	3298.36
Temperature/K	100.0
Crystal system	tetragonal
Space group	$I4_1/acd$
a/Å	31.4833(8)
b/Å	31.4833(8)
c/Å	35.7073(11)
α/°	90
β/°	90
γ/°	90
Volume/Å^3	35393(2)
Z	8
ρ_{calc} / g/cm^3	1.238
μ/mm^{-1}	3.329
F(000)	13056.0
Crystal size/mm^3	0.461 × 0.291 × 0.11
Radiation	MoKα (λ = 0.71073)
2Θ range for data collection/°	4.482 to 52.166
Index ranges	$-38 \leq h \leq 38$, $-38 \leq k \leq 38$, $-44 \leq l \leq 44$
Reflections collected	549070
Independent reflections	8768 [R_{int} = 0.0973, R_{sigma} = 0.0163]
Data/restraints/parameters	8768/0/397
Goodness-of-fit on F^2	1.048
Final R indexes [$I \geq 2\sigma(I)$]	R_1 = 0.0277, wR_2 = 0.0700
Final R indexes [all data]	R_1 = 0.0376, wR_2 = 0.0762
Largest diff. peak/hole /e Å^{-3}	0.63/–0.73

S4.2 Single-crystal X-ray Data Structure Refinement, and Structure Analysis for $1_{[5]}$···corannulene

Colourless crystals were grown via slow evaporation from an *ortho*-dichlorobenzene solution.

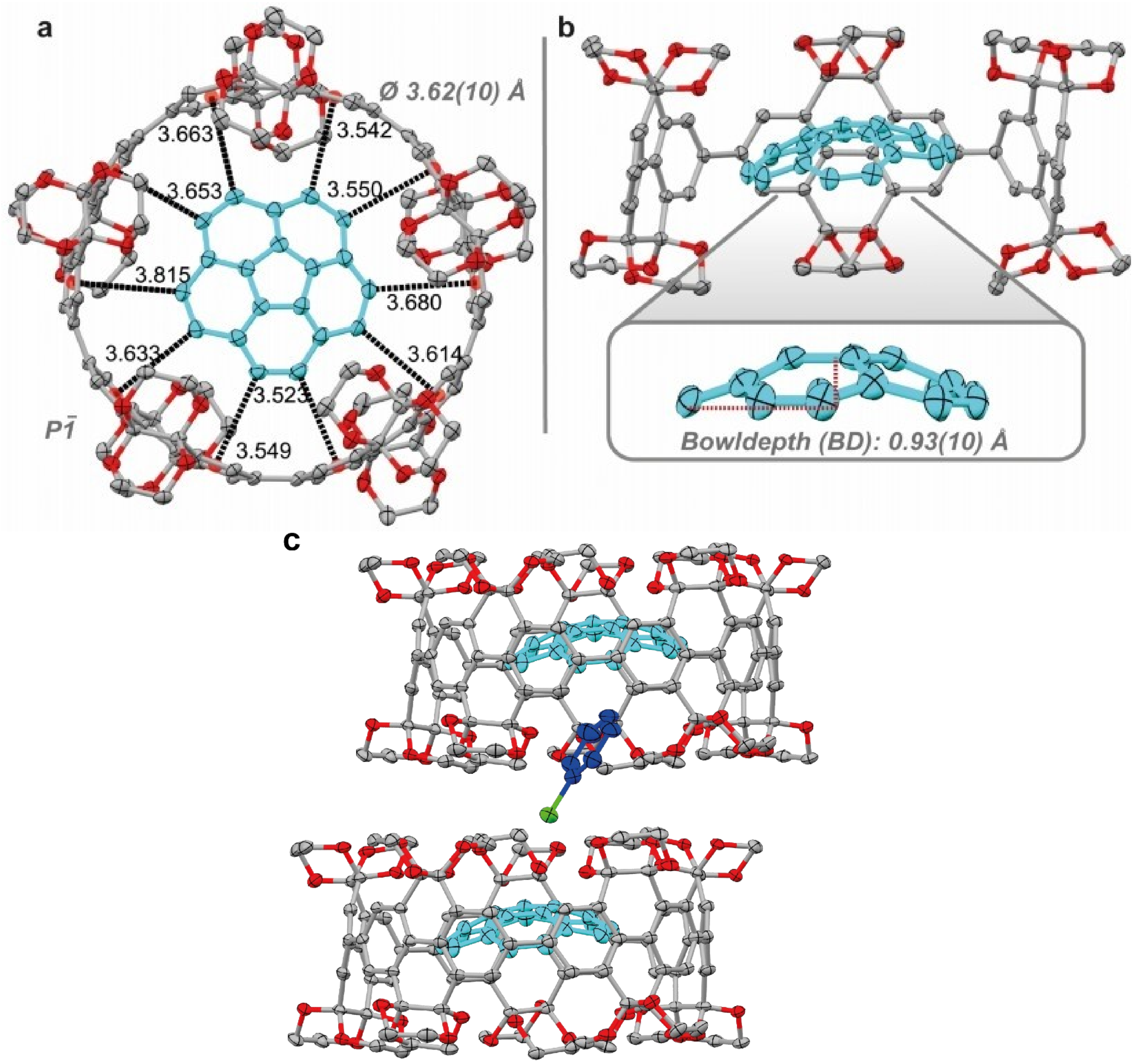

Figure S12 X-ray structure a) top view b) size view showing bowl depth of corannulene and c) crystal packing of **$1_{[5]}$···corannulene** at 100 K. Ellipsoids are shown at 50% probability, hydrogen atoms and solvent molecules are omitted for clarity. Colour code: carbon, grey; oxygen; red.

Table S2 Crystal data and structure refinement for $\mathbf{1_{[5]}}$**···corannulene**

CCDC Deposition number	236136
Empirical formula	$C_{146}H_{115} \cdot ClO_{40}$
Formula weight	2543.44
Temperature/K	100
Crystal system	triclinic
Space group	*P*-1
a/Å	11.072(2)
b/Å	19.137(4)
c/Å	34.621(7)
α/°	92.89(3)
β/°	95.52(3)
γ/°	99.88(3)
Volume/Å^3	7177(3)
Z	2
ρ_{calc}/g/cm^3	1.177
μ/mm^{-1}	0.104
F(000)	2655.0
Crystal size/mm^3	0.325 × 0.203 × 0.096
Radiation	MoKα (λ = 0.71073)
2Θ range for data collection/°	1.184 to 58.376
Index ranges	–15 ≤ h ≤ 15, –25 ≤ k ≤ 25, –47 ≤ l ≤ 47
Reflections collected	118286
Independent reflections	34072 [R_{int} = 0.0634, R_{sigma} = 0.0580]
Data/restraints/parameters	34072/232/1749
Goodness-of-fit on F^2	1.032
Final *R* indexes [$I \geq 2\sigma$ (I)]	R_1 = 0.0741, wR_2 = 0.2165
Final *R* indexes [all data]	R_1 = 0.1035, wR_2 = 0.2377
Largest diff. peak/hole / e Å^{-3}	0.83/–0.71

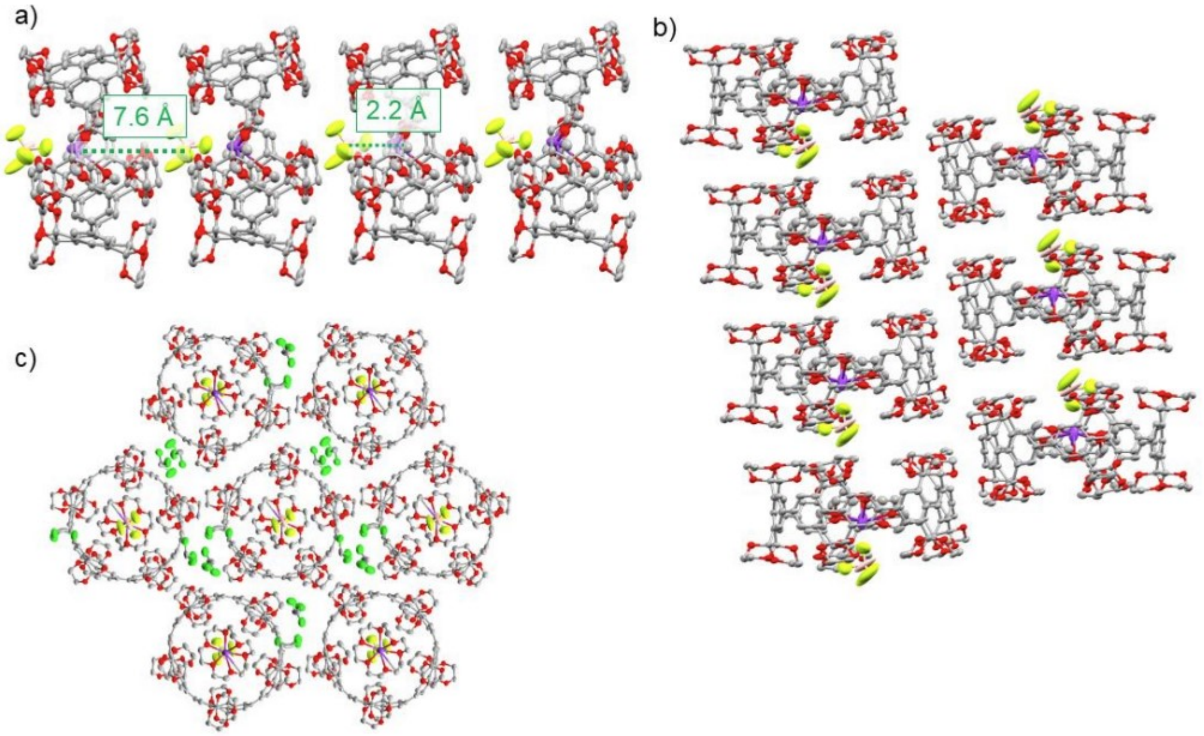

Figure S13 a) Previously reported[9] columnar packing of $\mathbf{1}_{[5]}\cdots$18-crown-6·K(BF_4) without solvent, showing the distances between the K^+ cation and the BF_4^- anion b) anti-parallel packing of two neighbouring columns c) cross-section through columnar packed $\mathbf{1}_{[5]}\cdots$18-crown-6·K(BF_4) with solvent molecule ($CHCl_3$).

S5. Geometrical Considerations and Volume Analysis of the Host $\mathbf{1}_{[5]}$

The cavity volume of $\mathbf{1}_{[5]}$ was calculated to be 480 Å^3 using the MS-Roll interface of X-Seed (Version 4.04),[13] employing the default van der Waals atomic radii and a probe sphere with a radius of 1.2 Å as suggested by the developer.[14] For $\mathbf{1}_{[5]}$ one coronene molecule was placed on the top and the bottom to prevent the probe exiting the cavity. The following default van der Waals radii were used: C = 1.70 Å, H = 1.20 Å, and O = 1.52 Å. The cavity shown was imaged using the POV-Ray interface of X-Seed.

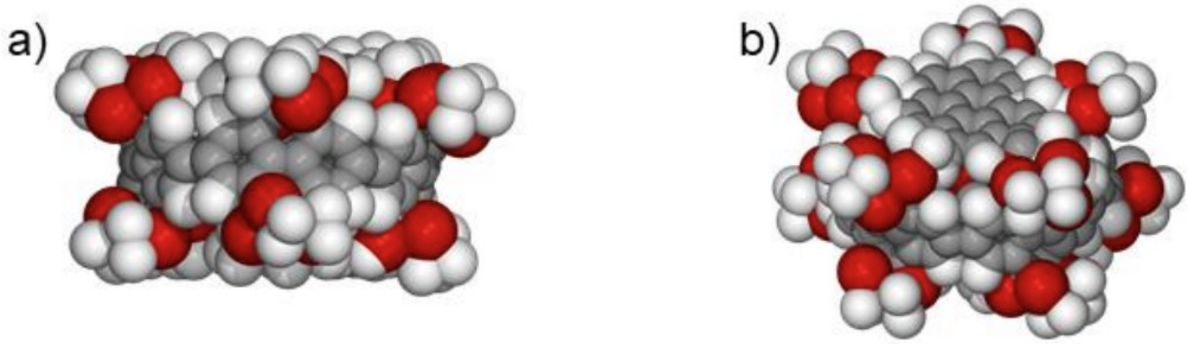

Figure S14 Van der Waals spheres a) side view and b) top view of $\mathbf{1}_{[5]}$ with a modelled corannulene placed on top and bottom of $\mathbf{1}_{[5]}$.

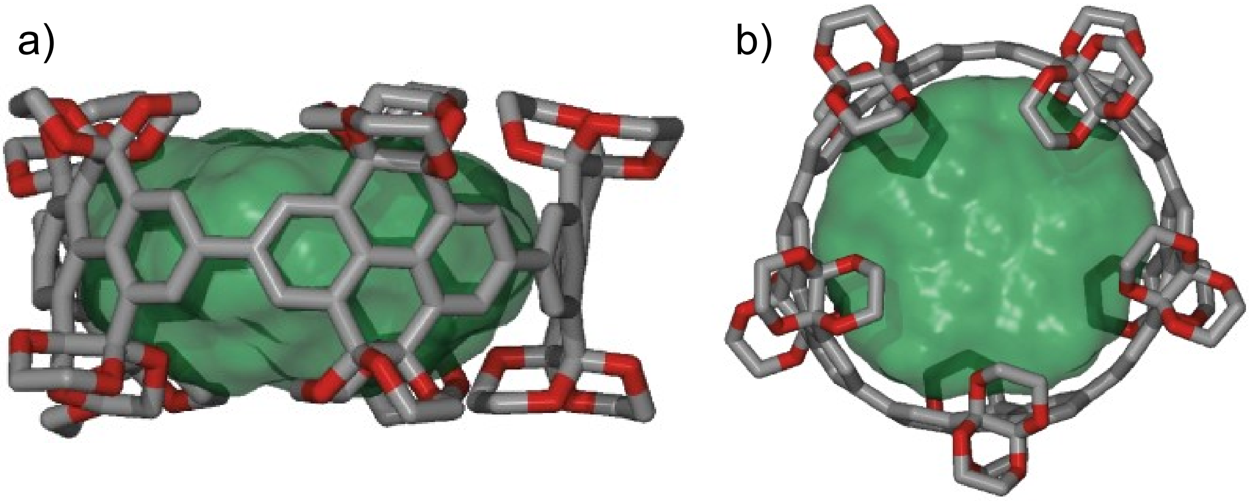

Figure S15 Ball and stick representation of the single-crystal X-ray structures a) side view and b) top view of $\mathbf{1}_{[5]}$ with the respective cavity volume calculated of 480 Å^3 using MS Roll (hydrogen atoms removed for clarity).

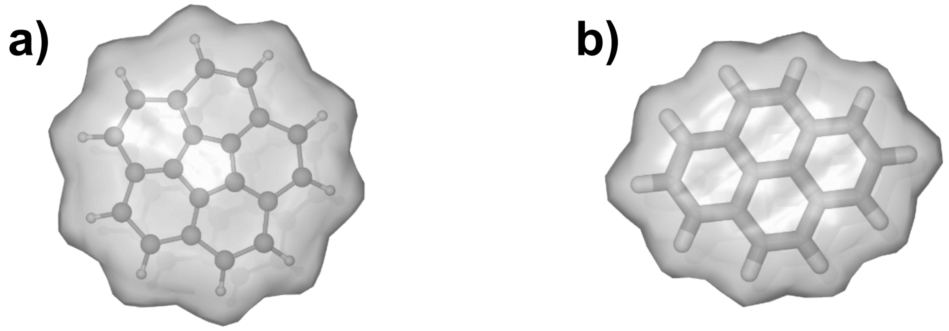

Figure S16 Graphical representation of the molecular volume calculated using the MS Roll suite of X–Seed, with a probe radius of 1.2 Å of a) corannulene (**9**), extracted from the crystal structure of $\mathbf{1}_{[5]}$**···corannulene** V = 224 Å^3, b) pyrene (**10**), geometry optimised structure at the B3LYP-D3/def2QZVP level of theory V = 191 Å^3.

Earlier work on packing coefficients found (empirically) that a packing coefficient is best in the range of (55±9)% for unpolar cavities (Mecozzi–Rebek rule).[15] The shown results for binding corannulene (**9**) and pyrene (**10**) are lower than that range. The deviation might be explained by polar interactions between host and guest and the ethylene glycol groups confining the cavity.

Table S3 Calculated host $\mathbf{1}_{[5]}$ and guest volumes with the respective packing coefficients

	V(guest) [Å^3]	*V*(cavity of $\mathbf{1}_{[5]}$) [Å^3]	Packing Coefficient
Corannulene **(9)**	224		47%
Pyrene **(10)**	191		40%
Benzotrithiophene **(11)**	197	480	41%
Coronene **(12)**	272		57%
Perylene **(13)**	237		49%
Octahydrotrindene **(14)**	215		45%

S6. Host-guest Binding Experiments by ^{1}H NMR Titration with $\mathbf{1}_{[5]}$

Titrations were performed at 298 K to determine the association constant (K_a) of the complexation between different host and guest molecules.

Preparation of Solutions. A solution of host ($\mathbf{1}_{[5]}$) (~ 0.2–0.5 mM) in an appropriate solvent (e. g. benzene-d_6, tetrachloroethane-d_2) was prepared first. This was then used as a parent solution to prepare a second solution as a mixture of host and guest. The constant component during the titration is referred to as "host" and the varied component as "guest" throughout.

Association Constant Determination. A solution of a known concentrations of guest $[G]_0$ (excess) with $[H]_0$ was added successively to the parent solution of $[H]_0$. To achieve higher addition of guest. Solid guest was added for the last additions if stated.

Distinct peaks emerged upon the addition of **corannulene** (**9**) (guest) to a solution of $\mathbf{1}_{[5]}$ (host)—this is indicative of slow exchange (see Figure S6.1 and S6.3). Therefore, the association constant (K_a) was determined using equation:

$$K_a = \frac{[HG]}{([H]_0 - a \times [HG])^a - ([G]_0 - b \times [HG])^b}$$

where [H] is the host concentration, [G] is the guest concentration, a is the host stoichiometry, b is the guest stoichiometry, and [HG] is the host-guest complex concentration determined by the following equation:

$$[HG] = \frac{n}{n+m} \times \frac{[H]_0}{a}$$

with a host-guest ratio $\left(\frac{n}{n+m}\right)$ chosen as close to 0.5 to minimise errors.[16],[18]

Table S4 Determination of association constants (K_a) of the complexation of $\mathbf{1}_{[5]}$ with **corannulene** in various deuterated solvents at 298 K.

**Solvent**	**Guest Equiv.**	$[H]_0$ **(mM)**	***n* / (*m* + *n*)**	K_a **(M^{-1})**	**Averaged** K_a **(M^{-1})**
Benzene-d_6	3.62	3.16×10^{-4}	0.543	1221	990
	4.47	2.85×10^{-4}	0.525	983	
	5.31	2.60×10^{-4}	0.490	769	
Tetrachloroethane-d_2	3.29	3.29×10^{-4}	0.409	730	622
	4.09	3.22×10^{-4}	0.405	574	
	5.09	3.13×10^{-4}	0.469	610	
	6.08	3.05×10^{-4}	0.494	573	
1:1 Toluene-d_8 / Tetrachloroethane-d_2	0.94	9.32×10^{-4}	0.396	1299	1140
	1.41	9.15×10^{-4}	0.459	982	

Upon addition of **pyrene** (**10**) (guest) to a solution of $\mathbf{1}_{[5]}$ (host), four sets of protons ($\mathbf{1}_{[5]}$ singlet and all three pyrene peaks) showed a significant change in chemical shift ($\Delta\delta$) at fast exchange (Figure S6.3). Therefore, the association constant (K_a) was determined by non-linear curve fitting (using the program bindfit and Origin 2019b) of the titration data according to the Benesi–Hildebrand method.[16] The fitting curves were obtained by plotting the change of chemical shift ($\Delta\delta$) of the host signals against the concentration of the guest and using the following equation: [16],[18]

$$\Delta\delta = \delta_{\Delta(HG)} \frac{\frac{1}{2} \times \left[\left([H]_{total} \times [G] + \frac{1}{K_a} \right) - \sqrt{\left([H]_{total} + [G] + \frac{1}{K_a} \right)^2 - (4 \times [H]_{total} \times [G])} \right]}{[H]_{total}}$$

where $\delta_{\Delta(HG)}$ is the chemical shift of the host-guest complex, and K_a is the association constant.

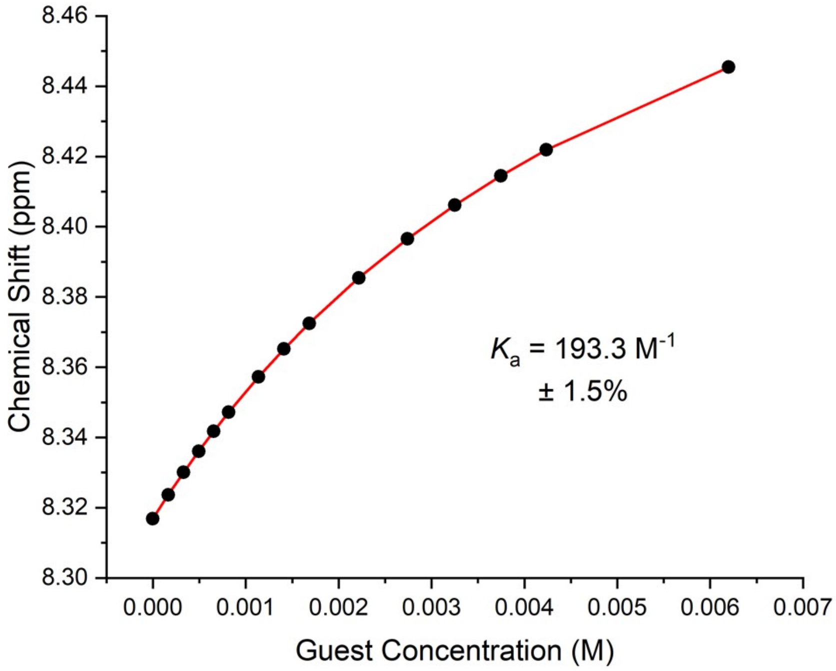

Figure S17 Non-linear least-square curve fitting analysis obtained from the isothermal binding titration of $\mathbf{1}_{[5]}$ (5.01 x 10^{-4} mol L^{-1} in C_6D_6 at 298 K) with 1:1 stoichiometry upon addition of **pyrene** (**10**). $\Delta\delta$ taken for $\mathbf{1}_{[5]}$ singlet. The association constant K_a was determined to be 193.3 M^{-1} ±1.5%. Including all experimental errors, the error is estimated to ±10%.

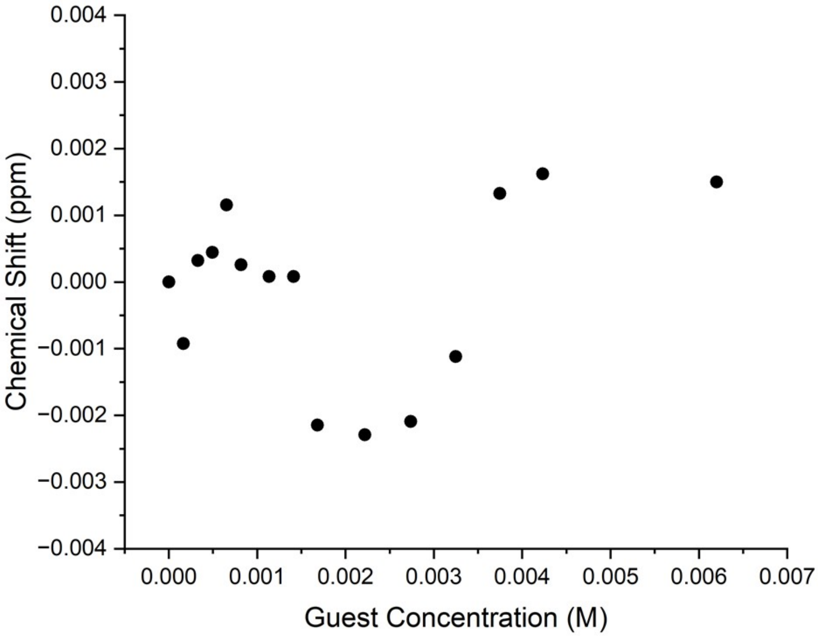

Figure S18 Residual difference plot of the predicted values of the fit *vs* experimental values of **$1_{[5]}$···pyrene**. Data acquired by isothermal binding titration with 1:1 stoichiometry.

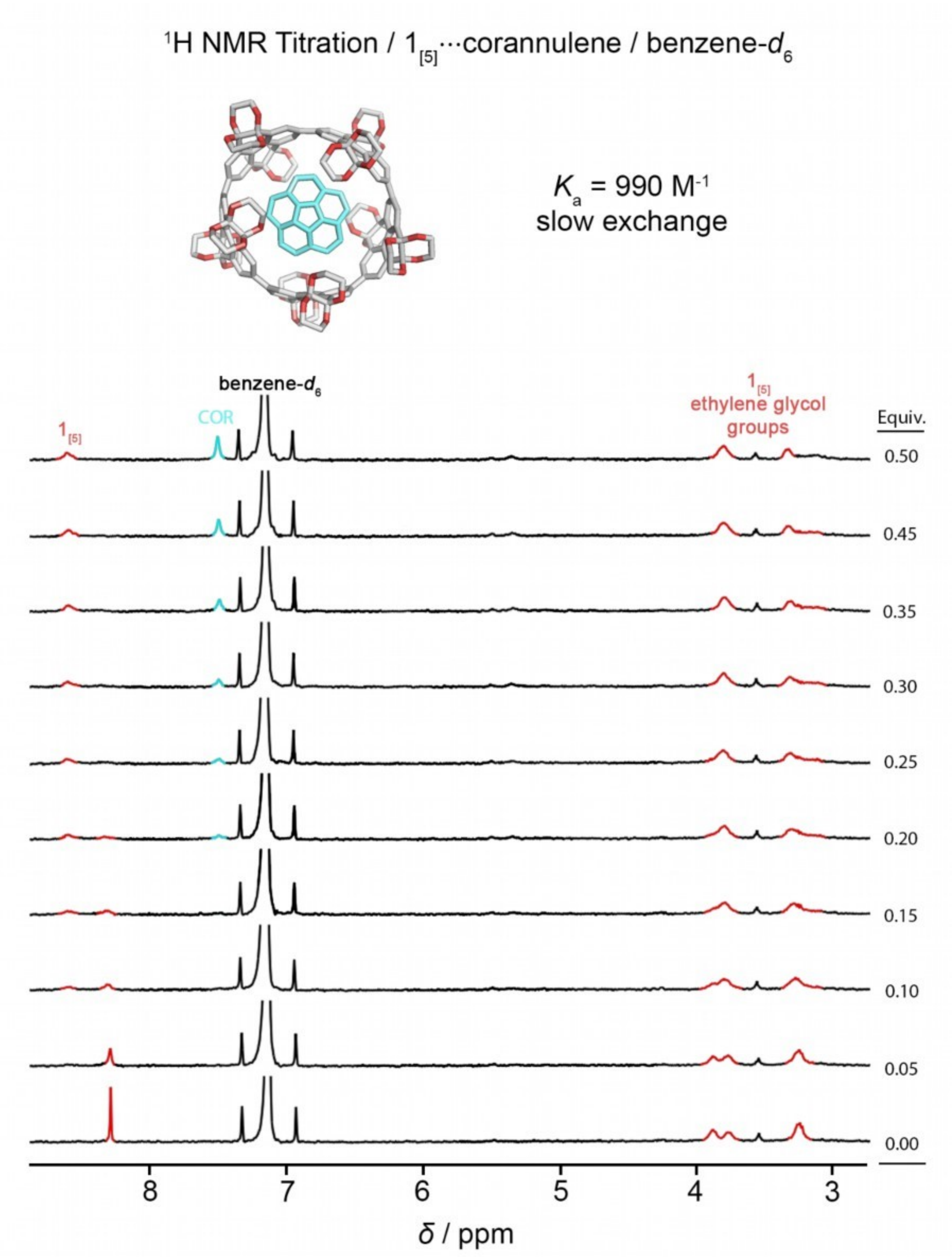

Figure S19 Stacked ^{1}H NMR spectra for the titration of a solution of $\mathbf{1_{[5]}}$ in benzene-d_6 with a solution of corannulene in benzene-d_6 at 298 K and 400 MHz. Colour code: $\mathbf{1_{[5]}}$, red; corannulene, blue.

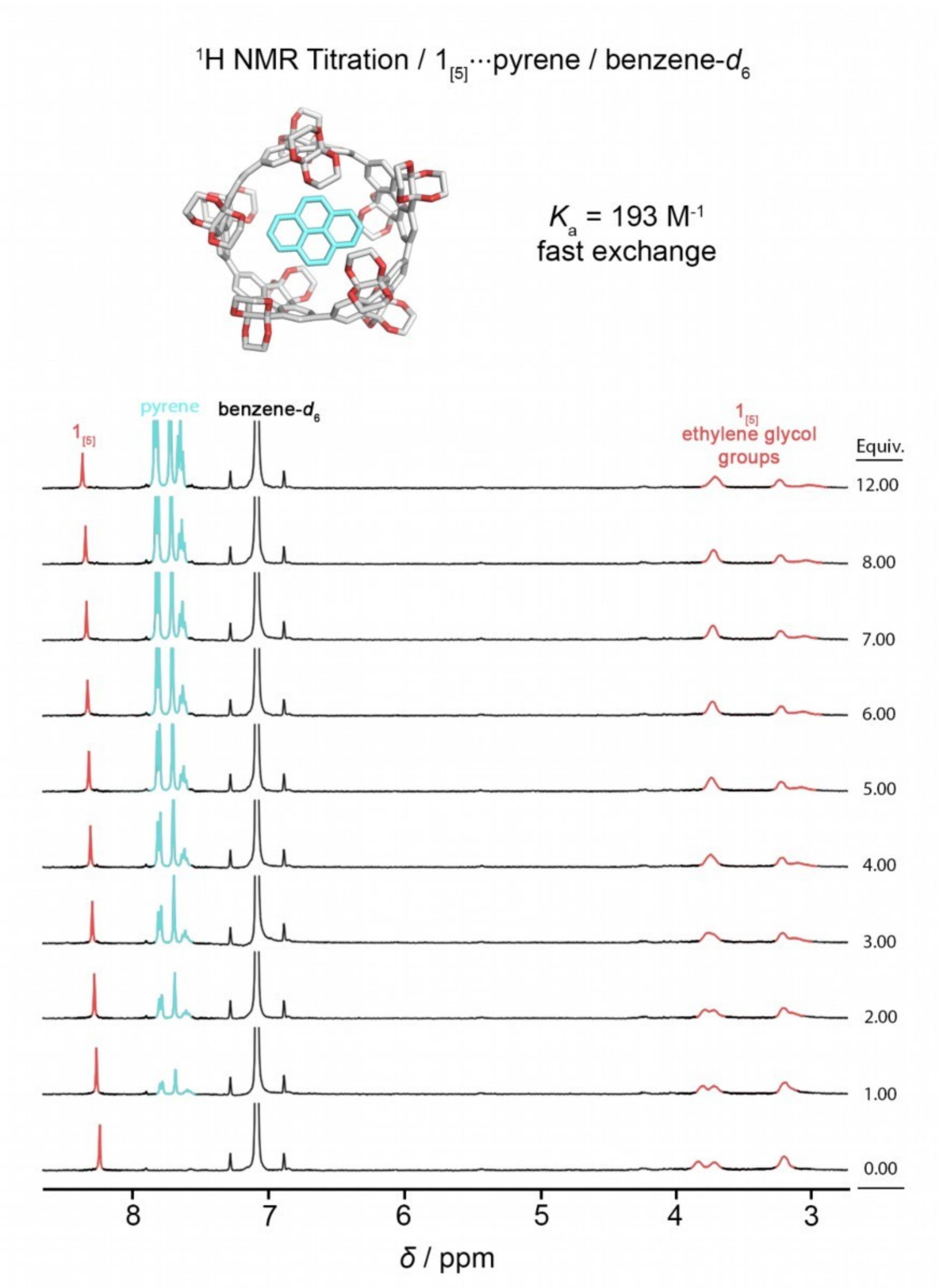

Figure S20 Stacked ^{1}H NMR spectra for the titration of a solution of $\mathbf{1}_{[5]}$ in benzene-d_6 with a solution of pyrene in benzene-d_6 at 298 K and 400 MHz. Colour code: $\mathbf{1}_{[5]}$, red; pyrene, blue.

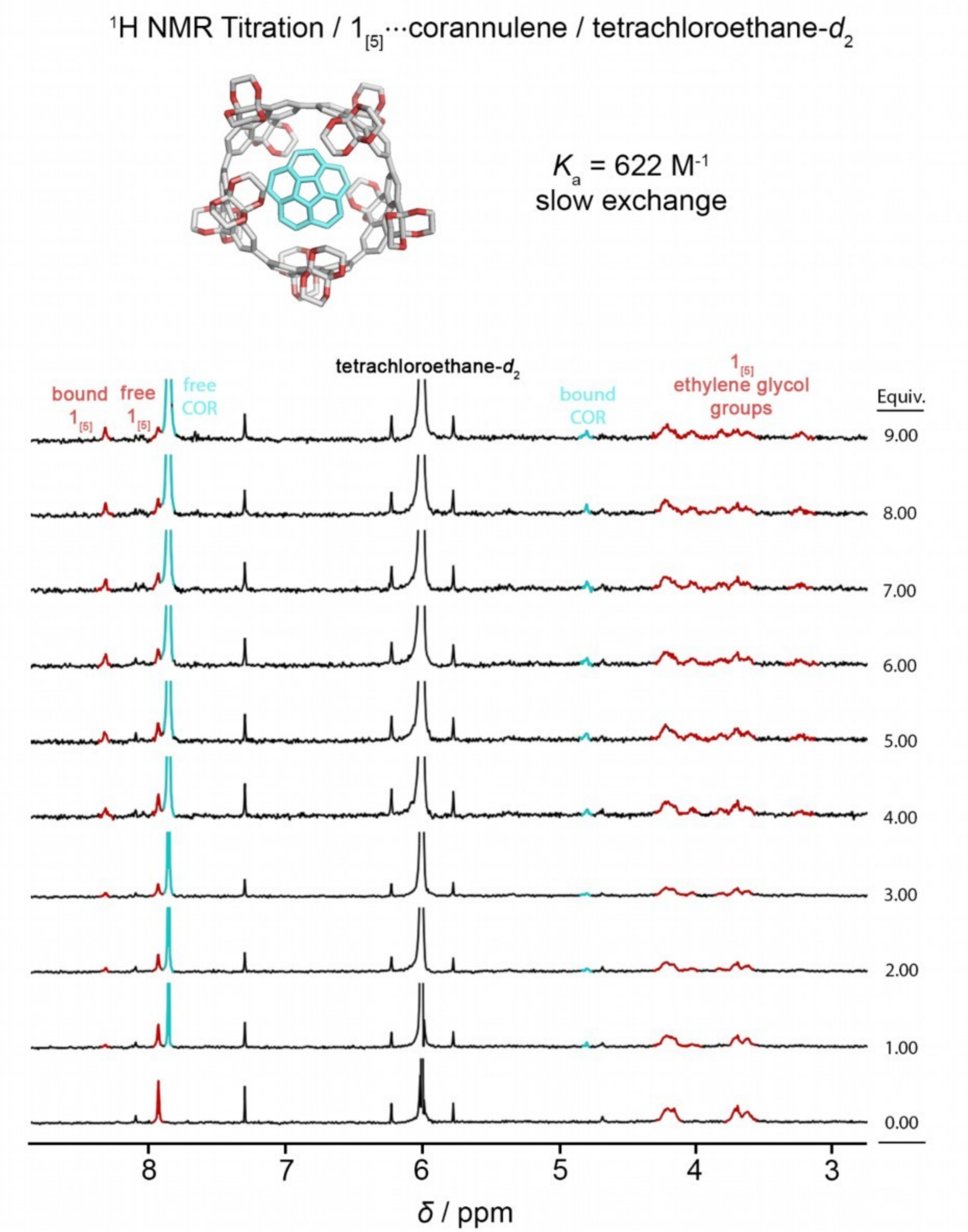

Figure S21 Stacked ^{1}H NMR spectra for the titration of a solution of $\mathbf{1}_{[5]}$ in tetrachloroethane-d_2 with a solution of corannulene in tetrachloroethane-d_2 at 298 K and 400 MHz. Colour code: $\mathbf{1}_{[5]}$, red; corannulene, blue. A relative population between free/bound host-guest complex was found to be to be 38:62 at 8 equivalence of corannulene at 298 K. This was calculated by creating a ratio of the integration between bound and free host peaks.

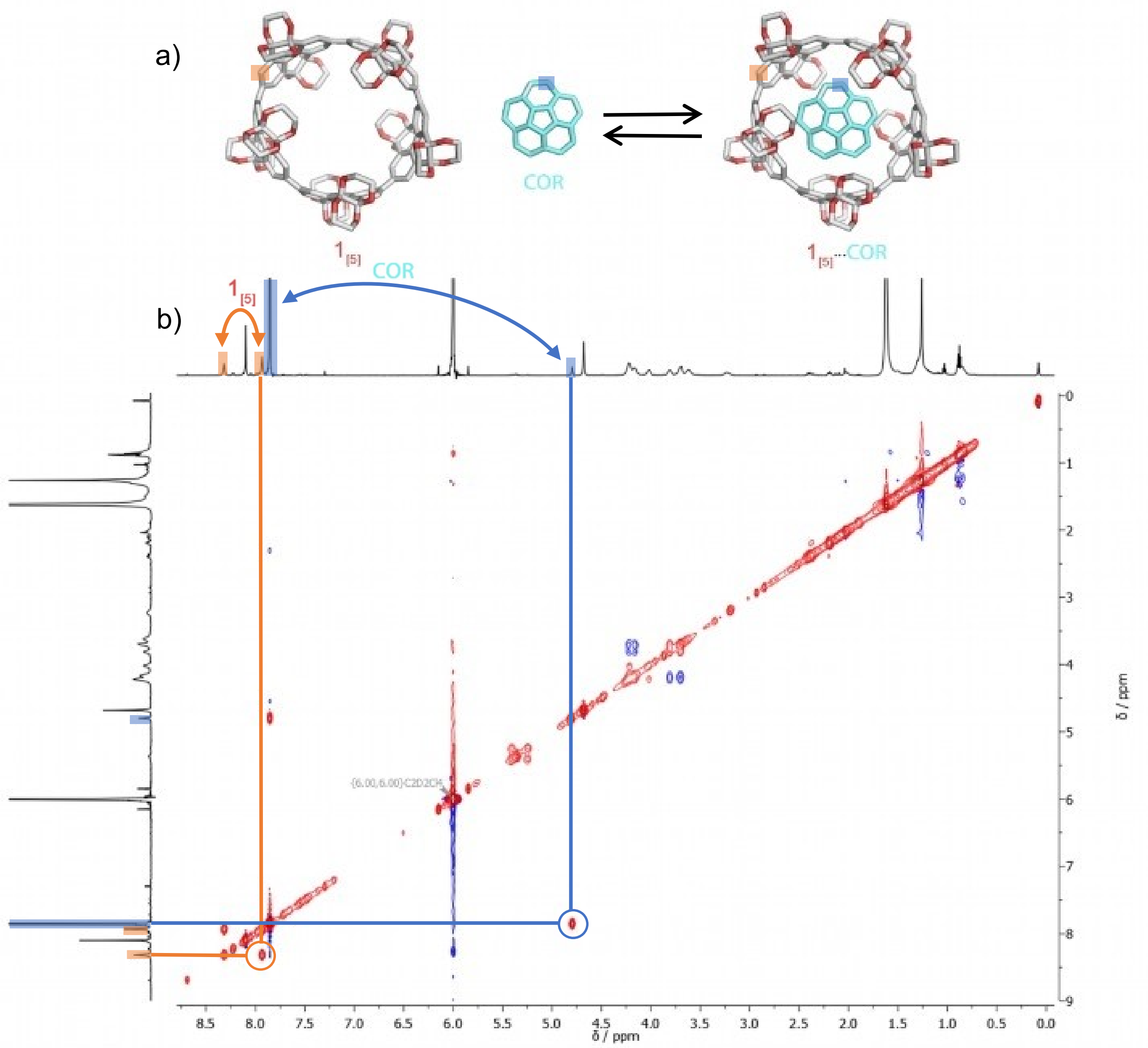

Figure S22 a) Scheme for the complexation between $\mathbf{1}_{[5]}$ and corannulene. **b)** ^{1}H-^{1}H ROESY ($C_2D_2Cl_4$, 600 MHz, 298 K) spectra of the complexation between $\mathbf{1}_{[5]}$ and corannulene with host-guest exchange crosspeaks highlighted.

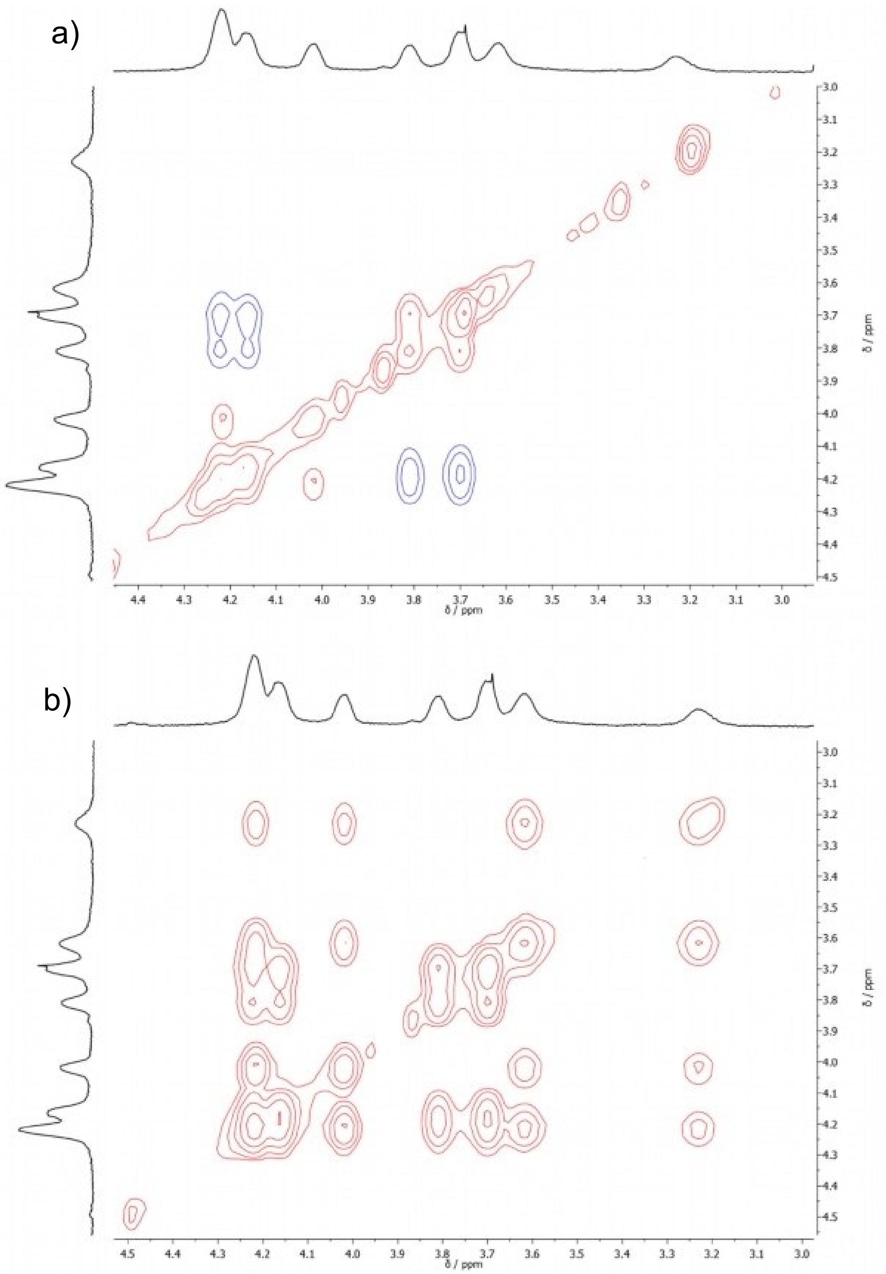

Figure S23 a) ^{1}H-^{1}H ROESY ($C_2D_2Cl_4$, 600 MHz, 298 K) spectra of the complexation between $\mathbf{1}_{[5]}$ and corannulene, zoomed in on the ethylene glycol group peaks (δ = 4.4–3.0 ppm). Red crosspeaks show chemical exchange of $\mathbf{1}_{[5]}$ from 'free' ($\mathbf{1}_{[5]}$) to 'bound' ($\mathbf{1}_{[5]}\cdots$COR). Blue crosspeaks show spatial proximity between equatorial inner and equatorial outer $\mathbf{1}_{[5]}$ protons (δ = 4.3–4.1 ppm) and axial inner and axial outer $\mathbf{1}_{[5]}$ protons (δ = 3.9–3.6 ppm). **b)** ^{1}H-^{1}H NOESY ($C_2D_2Cl_4$, 600 MHz, 298 K) spectra of the complexation between $\mathbf{1}_{[5]}$ and corannulene, zoomed in on the ethylene glycol group peaks (δ = 4.4–3.0 ppm). Red crosspeaks show spatial proximity between axial/equatorial inner $\mathbf{1}_{[5]}$ protons and axial/equatorial outer $\mathbf{1}_{[5]}$ protons. ^{1}H-^{1}H NOESY included as more sensitive than ^{1}H-^{1}H ROESY, however does not show chemical exchange crosspeaks due to large size of $\mathbf{1}_{[5]}$ (>2000 Da).

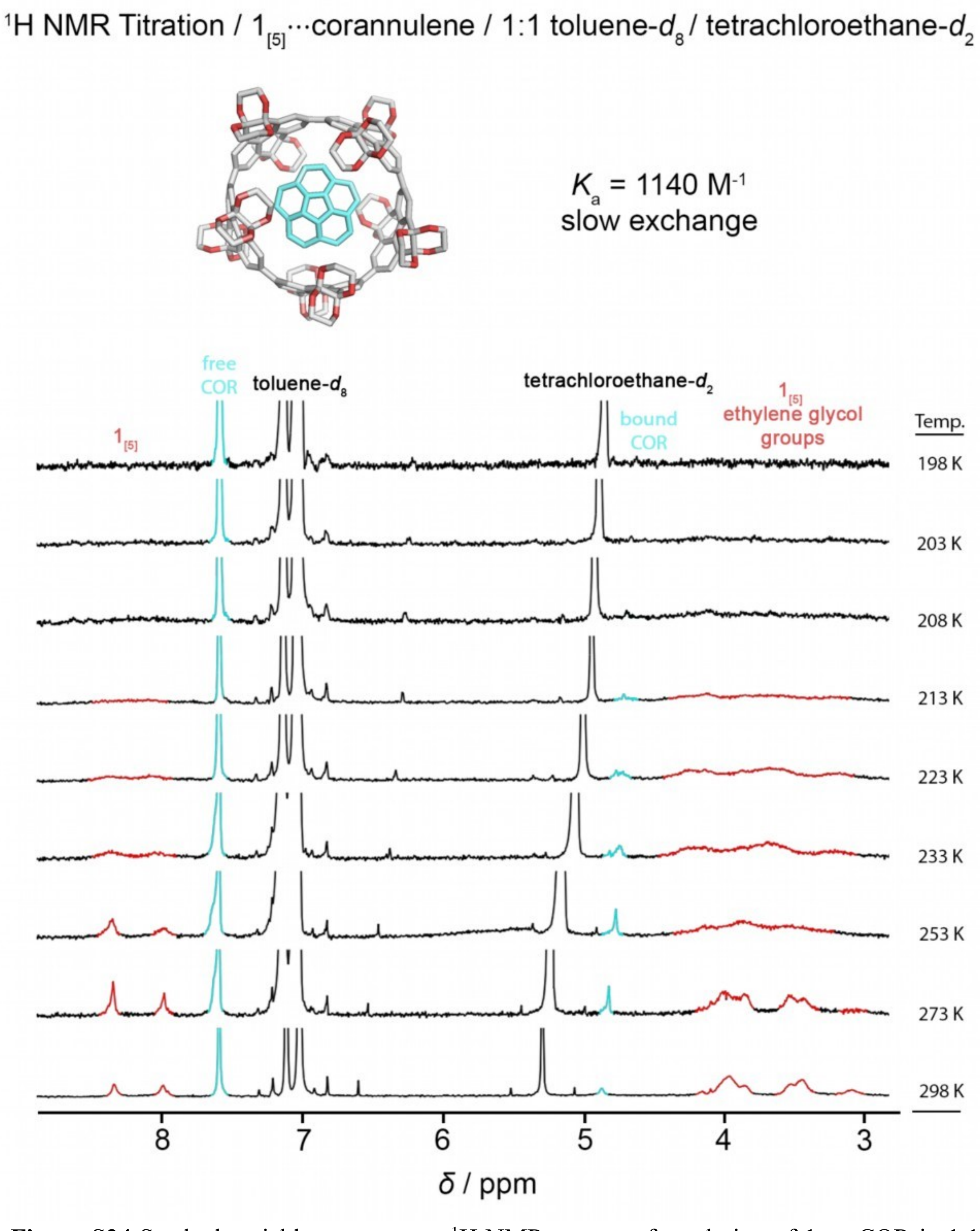

Figure S24 Stacked variable temperature ^{1}H NMR spectra of a solution of $\mathbf{1}_{[5]}$···COR in 1:1 toluene-d_8 / tetrachloroethane-d_2 at 298 K and 400 MHz. Colour code: $\mathbf{1}_{[5]}$, red; corannulene, blue. Note: $\mathbf{1}_{[5]}$ and bound corannulene signals disappear at temperatures below 213 K due to high viscosity of cold deuterated solvent mixture approaching its freezing point.

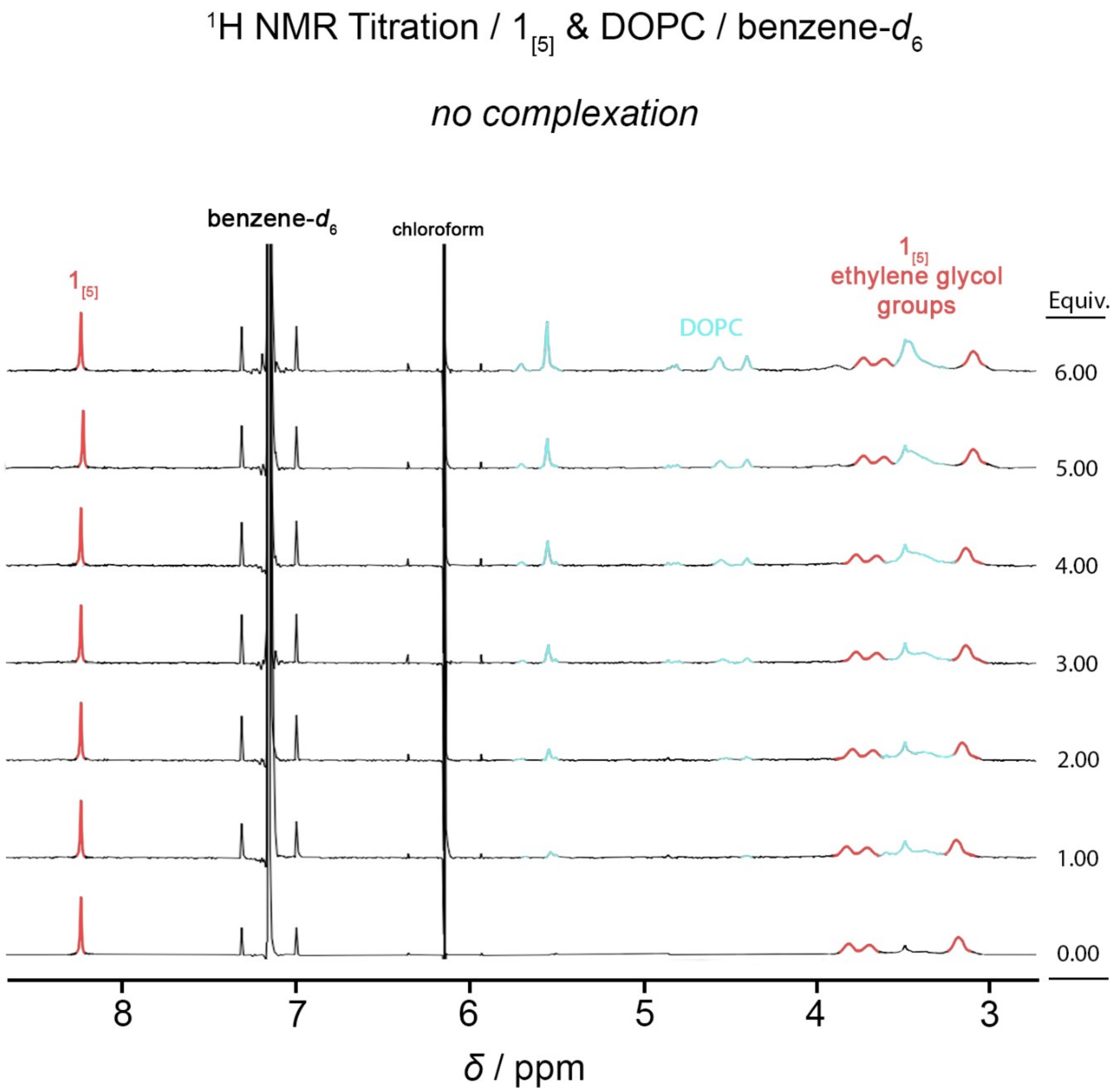

Figure S25 Stacked ^{1}H NMR spectra for the titration of a solution of $\mathbf{1}_{[5]}$ in benzene-d_6 with a solution of DOPC in benzene-d_6 at 300 K and 500 MHz. Colour code: $\mathbf{1}_{[5]}$, red; DOPC, blue.

S7. Preparation of vesicles

S7.1 Preparation of giant unilamellar vesicles (GUVs) for microscopic observations

A $CHCl_3$/MeOH (2:1, v/v) solution of 1,2-dioleoyl-*sn*-glycero-3-phosphocholine (DOPC, 1.0 mM), glucose (1.0 mM), and $\mathbf{1}_{[n]}$ (5.0 μM, n = 4–7) in a glass test tube was gently dried under Ar flow to form a thin lipid film. The film was dried under vacuum for 3 h at room temperature and then pre-hydrated with deionised H_2O (5.0 μL) for 10 min at 55 °C. Deionised H_2O (195 μL) was added and hydrated for 16 h at 55 °C to obtain a dispersion of DOPC GUVs ([DOPC] = 200 µM, $\mathbf{1}_{[n]}$ (n = 4–7) = 1.0 μM, [glucose] = 200 µM). For the preparation of GUVs containing phosphatidylethanolamine (PE) or phosphatidylserine (PS), [PC]/[PE] = 80/20 ([total lipid] = 1.0 mM) or [PC]/[PS] = 80/20 ([total lipid] = 1.0 mM) solution was used and the GUVs were prepared according to the same protocol as described above.

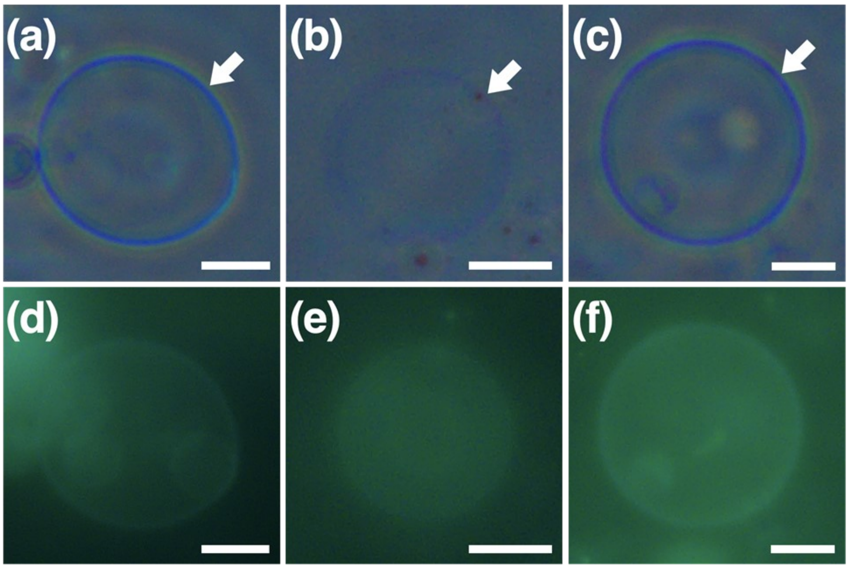

Figure S26 (a–c) Phase contrast and (d–f) fluorescence micrographs (λ_{ex} = 340–390 nm, λ_{obsd} > 420 nm) of LUVs containing (a, d) PC, (b, e) PE, and (c, f) PS ([total lipid] = 200 μM) in aqueous glucose ([glucose] = 200 μM) in the presence of $\mathbf{1}_{[5]}$ ([$\mathbf{1}_{[5]}$] = 1 μM) at 25 °C. Scale bars: 10 μm.

S7.2 Preparation of large unilamellar vesicles (LUVs) for absorption spectroscopy

A $CHCl_3$ solution (1.0 mL) of DOPC and $\mathbf{1}_{[5]}$ ([DOPC] = 10 mM, $\mathbf{1}_{[5]}$ = 50 μM) in an amber test tube was slowly evaporated to dryness using a rotary evaporator at room temperature, and the resulting film was further dried under vacuum overnight. A lipid film developed on the

inner surface of the test tube was then hydrated with a HEPES buffer ([HEPES] = 20 mM, [NaCl] = 50 mM, pH 7.1, 1.0 mL) for 1 h at 37 °C, and the mixture was vortexed for 1 min. After 5 freeze-to-thaw cycles, the resulting mixture was extruded through a porous polycarbonate membrane with a pore diameter of 100 nm for 21 times at room temperature to obtain a dispersion of DOPC LUVs in HEPES buffer ([DOPC] = 10 mM, $\mathbf{1}_{[5]}$ = 50 μM, [HEPES] = 20 mM, [NaCl] = 50 mM, pH 7.1).

S7.3 Preparation of LUVs for fluorescence depth quenching experiments

A $CHCl_3$ solution (1.0 mL) of DOPC, 16:0-12 doxyl PC (doxyl PC), and $\mathbf{1}_{[5]}$ ([total PC] = 1.0 mM, [doxyl PC]/[DOPC] = 0:100, 2.5:97.5, 5:95, or 10:90, [$\mathbf{1}_{[5]}$] = 5.0 μM) in an amber test tube was slowly evaporated to dryness using a rotary evaporator at room temperature, and the resulting film was further dried under vacuum overnight. A lipid film developed on the inner surface of the test tube was then hydrated with a HEPES buffer ([HEPES] = 20 mM, [NaCl] = 50 mM, pH 7.1, 1.0 mL) for 1 h at 37 °C, and the mixture was vortexed for 1 min. After five freeze-to-thaw cycles, the resulting mixture was extruded through a porous polycarbonate membrane with a pore diameter of 100 nm for 21 times at room temperature to obtain a dispersion of LUVs in HEPES buffer ([total PC] = 1.0 mM, [doxyl PC]/[DOPC] = 0/100, 2.5/97.5, 5/95, or 10/90, [$\mathbf{1}_{[5]}$] = 5.0 μM).

S7.4 Preparation of LUVs for ion transport assays

A $CHCl_3$ solution (1.0 mL) of DOPC and the mixture of $\mathbf{1}_{[n]}$ (n = 4–7) ([DOPC] = 10 mM, [$\mathbf{1}_{[n]}$] = 120 μg mL^{-1}) in an amber test tube was slowly evaporated to dryness using a rotary evaporator at room temperature, and the resulting film was further dried under vacuum overnight. A lipid film developed on the inner surface of the test tube was then hydrated with a HEPES buffer ([HEPES] = 20 mM, [NaCl] = 50 mM, [HPTS] = 3.0 mM, pH 7.1, 1.0 mL) for 1 h at 37 °C, and the mixture was vortexed for 1 min. After 5 freeze-to-thaw cycles, the resulting mixture was extruded through a porous polycarbonate membrane with a pore diameter of 100 nm for 21 times at room temperature to obtain a dispersion of DOPC LUVs in HEPES buffer ([DOPC] = 10 mM, [$\mathbf{1}_{[n]}$] = 120 μg mL^{-1}, [HEPES] = 20 mM, [NaCl] = 50 mM, [HPTS] = 3.0 mM, pH 7.1). The obtained suspension of LUVs was dialysed at 4 ºC in a HEPES buffer ([HEPES] = 20 mM, [NaCl] = 50 mM, pH 7.1) using Spectra/Por Dialysis Membrane (MWCO 3500).

S7.5 Ion transport assays using LUVs

To a stirred solution of HEPES buffer ([HEPES] = 20 mM, [NaCl] = 50 mM, [HPTS] = 3.0 mM, pH 7.1, 1948 μL) was added a HEPES buffer dispersion ([HEPES] = 20 mM, [NaCl] = 50 mM, pH 7.1, 20 μL) of LUVs containing HPTS ([DOPC] = 10 mM, [$\mathbf{1}_{[n]}$] = 120 μg mL^{-1}, [HPTS] = 3.0 mM). Then, an aqueous MOH solution ([MOH] = 600 mM, M = Li, Na, K, 32 μL) was added to the mixture using a syringe pump under dark at 25 °C. After 300 s, aqueous Triton X-100 (10%, *v*/*v*, 30 μL) was added to the mixture for the lysis of LUVs. Throughout the experiment, fluorescence intensity of HPTS at 510 nm upon excitation by 460 nm light was monitored.

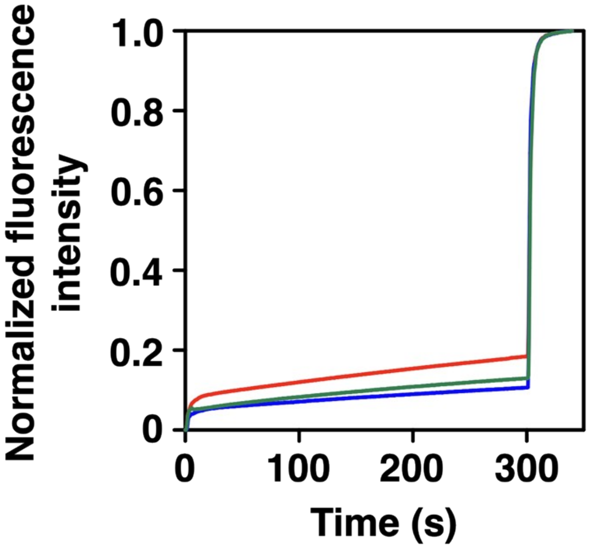

Figure S27 Time course changes of HPTS fluorescence intensity (λ_{em} =510 nm, λ_{ex}=460 nm) encapsulated in DOPC LUVs in a HEPES buffer upon addition of different MOH (M = Li (red), Na (blue), K (green)).

S7.6 Current recording measurement of $\mathbf{1}_{[5]}$-containing black lipid membranes

A trace amount of *n*-octane solution of DPhPC was deposited on an orifice (diameter: 100 μm) sandwiched by *cis* and *trans* chambers containing aqueous HCl ([HCl] = 100 mM) to prepare black lipid membranes. A trace amount of THF solution of $\mathbf{1}_{[5]}$ was added continuously under the application of voltage (+100 mV). The current profile was recorded at room temperature.

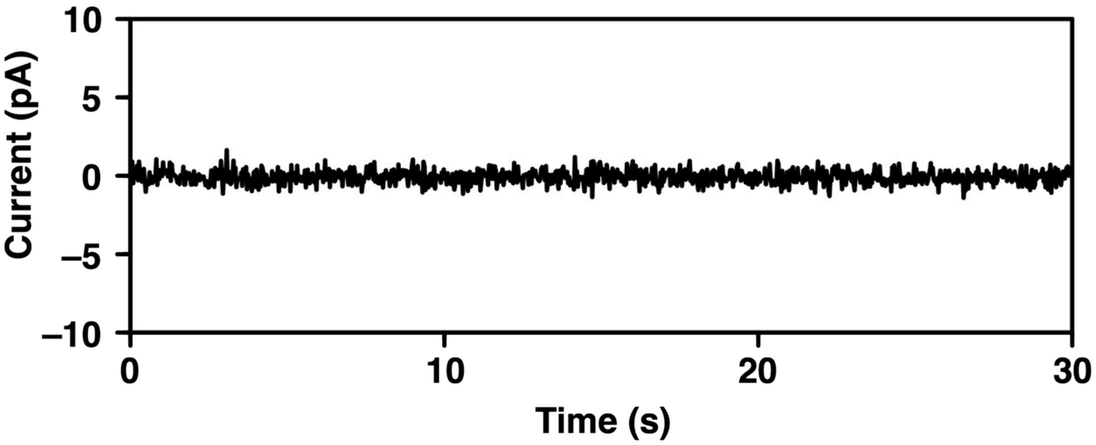

Figure S28 Current vs time plot of $\mathbf{1}_{[5]}$-containing black lipid membranes in aqueous HCl at an applied voltage of +100 mV at room temperature.

S8. MD simulations

Optimised structure of $\mathbf{1}_{[5]}$ was calculated by using B3LYP/6-31G(d) as implemented on Gaussian 16 a quantum chemistry package.[20] The bilayer membrane model consisted of 64 DOPC molecules per layer of leaflet. One $\mathbf{1}_{[5]}$ was embedded in the membrane and water molecules using the Membrane Builder implemented in CHARMM-GUI.[21–26] The center of gravity of $\mathbf{1}_{[5]}$ were placed at z = 0, 10, and 20 Å, with the z = 0 plane between the leaflets of the membrane. Five different initial models were built as shown in Figure S9. The prepared models were solvated using 6400 TIP3[27] water molecules and adequate numbers of Na^+ and Cl^- ions along the z-axis direction.

All-atom molecular dynamics (MD) simulations of $\mathbf{1}_{[5]}$ embedded in the membrane-water system were carried out. All the MD simulations were performed using the MD program package GROMACS ver. 2022.4.[28–30] The force field for the membranes and the solvent molecules was the CHARMM36 m force field,[31–35] and that for $\mathbf{1}_{[5]}$ was the CHARMM General Force Field (CGenFF).[36] The electrostatic interaction was handled by the smooth particle mesh Ewald method,[37] and the van der Waals interaction was truncated by the switching function with the range of 10–12 Å. The bond lengths involving hydrogen atoms were constrained by the P-LINCS algorithm.[38] Before the production runs, according to the default setups of the CHARMM-GUI, an energy minimisation and multi-step equilibration runs were executed sequentially. All simulations were run at 2 fs per step for a total of 1 μs in NPT ensemble at a temperature of 300 K and a pressure of 1.0 bar. The thermostat and the barostat were the Nose-Hoover scheme[39, 40] and the Parrinello-Rahman approach,[41, 42] respectively.

To examine the position of $\mathbf{1}_{[5]}$ in the membrane, the z-coordinates of the center of gravity of $\mathbf{1}_{[5]}$ were traced through MD trajectories. On the other hand, to examine the orientation of $\mathbf{1}_{[5]}$ in the membrane, the angle θ between the z-axis and the central axis of the decagonal prism with the 20 carbon atoms of $\mathbf{1}_{[5]}$ as vertices was calculated.

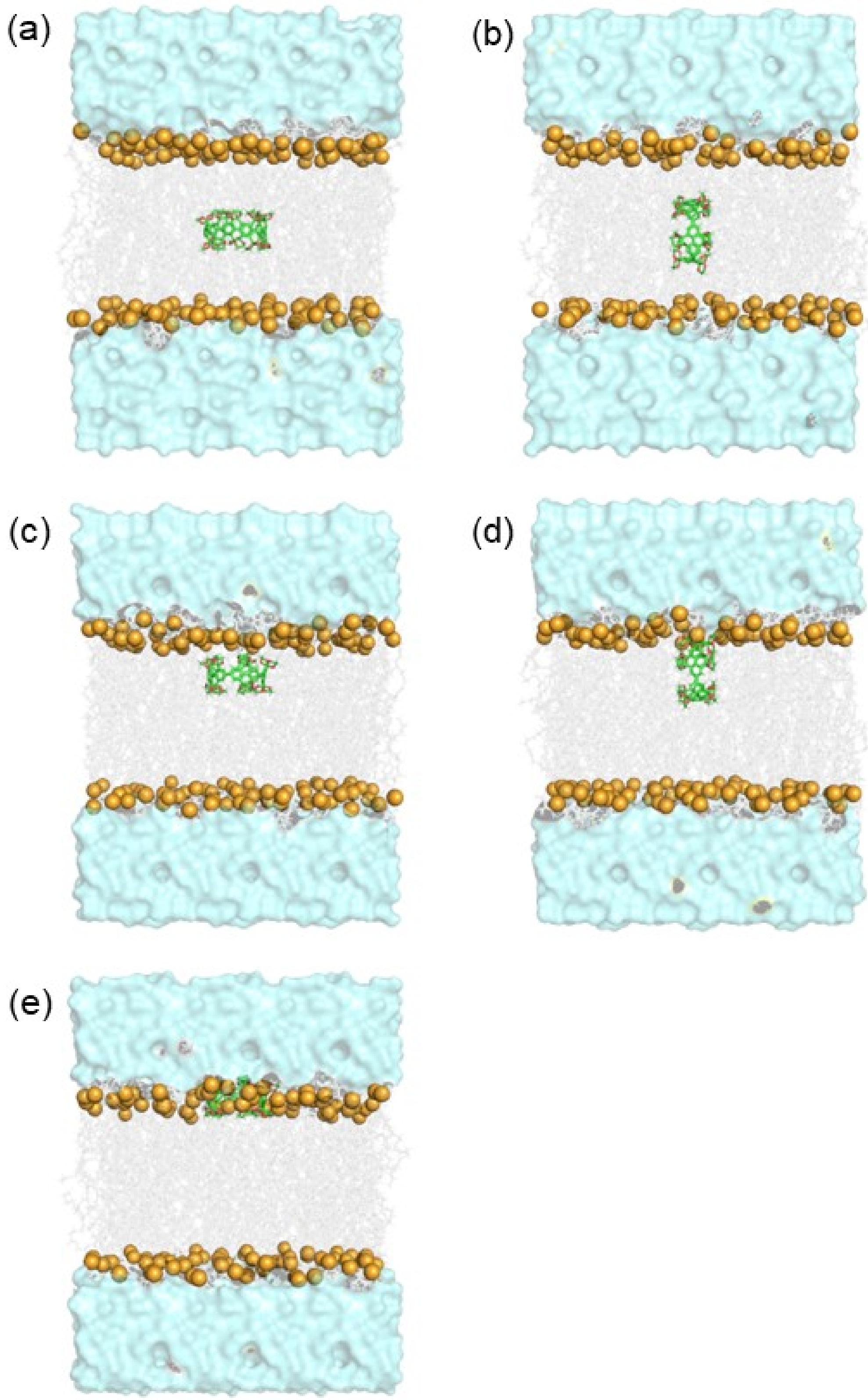

Figure S29 Initial configurations of $\mathbf{1}_{[5]}$ embedded in the membrane-water system for MD simulations. (a) z = 0 Å and θ = 0°, (b) z = 0 Å and θ = 90°, (c) z = 10 Å and θ = 0°, (d) z = 10 Å and θ = 90°, (e) z = 20 Å and θ = 0°.

S9. References

[1] G. M. Sheldrick, *Sadabs*, 1996.

[2] G. M. Sheldrick, *Acta Crystallogr. Sect. A*, 2015, **71**, 3–8.

[3] G. M. Sheldrick, *Acta Crystallogr. Sect. C,* 2015, **71**, 3–8.

[4] O. V. Dolomanov, L. J. Bourhis, R. J. Gildea, J. A. K. Howard and H. Puschmann, *J. Appl. Crystallogr*., 2009, **42**, 339–341.

[5] B. Rees, L. Jenner and M. Yusupov, *Acta Crystallogr. Sect. D Biol. Crystallogr*., 2005, **61**, 1299–1301.

[6] P. van der Sluis and A. L. Spek, *Acta Crystallogr. Sect. A,* 1990, **46**, 194–201.

[7] A. L. Spek, *J. Appl. Crystallogr*., 2003, **36**, 7–13.

[8] B. L. Hu, K. Zhang, C. An, D. Schollmeyer, W. Pisula and M. Baumgarten, *Angew. Chem. Int. Ed*., 2018, **57**, 12375–12379.

[9] N. Grabicki, K. T. D. Nguyen, S. Weidner and O. Dumele, *Angew. Chem. Int. Ed*., 2021, **60**, 14909–14914.

[10] J. Merz, M. Dietz, Y. Vonhausen, F. Wöber, A. Friedrich, D. Sieh, I. Krummenacher, H. Braunschweig, M. Moos, M. Holzapfel, C. Lambert and T. B. Marder, *Chem. Eur. J.,* 2020, **26**, 438–453.

[11] D. N. Coventry, A. S. Batsanov, A. E. Goeta, J. A. K. Howard, T. B. Marder and R. N. Perutz*, Chem. Commun*., 2005, 2172–2174.

[12] S. Hitosugi, W. Nakanishi, T. Yamasaki and H. Isobe, *Nat. Commun*., 2011, **2**, 492.

[13] L. J. Barbour, *J. Appl. Crystallogr*., 2020, **53**, 1141–1146.

[14] D. P. van Heerden and L. J. Barbour, *Chem. Soc. Rev*., 2021, **50**, 735–749.

[15] S. Mecozzi and J. Rebek, *Chem. Eur. J*., 1998, **4**, 1016–1022.

[16] D. Brynn Hibbert and P. Thordarson*, Chem. Commun*., 2016, **52**, 12792–12805.

[17] J. R. Long and R. S. Drago, *J. Chem. Educ*., 1982, **59**, 1037.

[18] P. Thordarson, *Chem. Soc. Rev*., 2011, **40**, 1305–1323.

[19] C. A. Schneider, W. S. Rasband, and K. W. Eliceiri, *Nature Methods*, 2012, **9**, 671–675.

[20] Gaussian 16, Revision C.01, M. J. Frisch, G. W. Trucks, H. B. Schlegel, G. E. Scuseria, M. A. Robb, J. R. Cheeseman, G. Scalmani, V. Barone, G. A. Petersson, H. Nakatsuji, X. Li, M. Caricato, A. V. Marenich, J. Bloino, B. G. Janesko, R. Gomperts, B. Mennucci, H. P. Hratchian, J. V. Ortiz, A. F. Izmaylov, J. L. Sonnenberg, D. Williams-Young, F. Ding, F. Lipparini, F. Egidi, J. Goings, B. Peng, A. Petrone, T. Henderson, D. Ranasinghe, V. G. Zakrzewski, J. Gao, N. Rega, G. Zheng, W. Liang, M. Hada, M. Ehara, K. Toyota,

R. Fukuda, J. Hasegawa, M. Ishida, T. Nakajima, Y. Honda, O. Kitao, H. Nakai, T. Vreven, K. Throssell, J. A. Montgomery, Jr., J. E. Peralta, F. Ogliaro, M. J. Bearpark, J. J. Heyd, E. N. Brothers, K. N. Kudin, V. N. Staroverov, T. A. Keith, R. Kobayashi, J. Normand, K. Raghavachari, A. P. Rendell, J. C. Burant, S. S. Iyengar, J. Tomasi, M. Cossi, J. M. Millam, M. Klene, C. Adamo, R. Cammi, J. W. Ochterski, R. L. Martin, K. Morokuma, O. Farkas, J. B. Foresman, and D. J. Fox, Gaussian, Inc., Wallingford CT, 2016.

[21] S. Jo, T. Kim, V. G. Lyer and W. Im, *J. Comput. Chem.,* 2008, **29**, 1859–1865.

[22] S. Jo, T. Kim and W. Im, *PLoS One*, 2007, **2**, e880.

[23] S. Jo, J. B. Lim, J. B. Klauda and W. Im, *Biophys. J.,* 2009, **97**, 50–58.

[24] E. L. Wu, X. Cheng, S. Jo, H. Rui, K. C. Song, E. M. Dávila-Contreras, Y. Qi, J. Lee, V. Monje-Galvan, R. M. Venable, J. B. Kauda and W. Im, *J. Comput. Chem.,* 2014, **35**, 1997–2004.

[25] J. Lee, X. Cheng, J. M. Swails, M. S. Yeom, P. K. Eastman, J. A. Lemkul, S. Wei, J. Buckner, J. C. Jeong, Y. Qi, S. Jo, V. S. Pande, D. A. Case, C. L. Brooks III, A. D. MakKerell Jr, J. B. Kauda and W. Im, *J. Chem. Theory Comput.,* 2016, **12**, 405–413.

[26] J. Lee, D. S. Paterl, J. Ståhle, S−J. Park, N. R. Kern, S. Kim, J. Lee, X. Cheng, M. A. Valvano, O. Holst, Y. Knirel, Y. Qui, S. Jo, J. B. Kauda, G. Widmalm and W. Im, *J. Chem. Theory Comput.,* 2019, **15**, 775–786.

[27] W. L. Jorgensen, J. Chandrasekhar, J. D. Madura, R. M. Impey and M. L. Klein, *J. Chem. Phys.,* 1983, **79**, 926–935.

[28] M. J. Abraham, T. Murtola, R. Schuls, S. Páll, J. C. Smith, B. Hess and E. Lindahl, *Software X,* 2015, **1**, 19–25.

[29] S. Páll, M. J. Abraham, C. Kutzner, B. Hess, E. Lindahl, in S. Markidis and E. Laure, *EASC*, 2015, **8759**, 3–27.

[30] S. Pronk, S. Páll, R. Schulz, P. Larsson, P. Bjelkmar, R. Apostolov, M. R. Shirts, J. C. Smith, P. M. Kasson, D. van der Spoel, B. Hess and E. Lindahl, *Bioinformatics,* 2013, **29**, 845–854.

[31] J. Huang, S. Rauscher, G. Nawrocki, T. Ran, M. Feig, B. L. de Groot, H. Grubmüller and A. D. MacKerell, *Nat. Methods,* 2017, **14**, 71–73.

[32] A. D. MacKerell, D. Bashford, M. Bellott, R. L. Dunbrack, J. D. Evanseck, M. J. Field, S. Fischer, J. Gao, H. Guo, S. Ha, D. Joseph-McCarthy, L. Kuchnir, K. Kuczera, F. T. Lau, C. Mattos, S. Michnick, T. Ngo, D. T. Nguyen, B. Prodhom, W. E. Reiher, B. Roux,

M. Schlenkrich, J. C. Smith, R. Stote, J. Straub, M. Watanabe, J. Wiórkiewicz-Kuczera, D. Yin and M. Karplus, *J. Phys. Chem. B.,* 1998, **102**, 3586–3616.

[33] A. D. MacKerell Jr, M. Feig and C. L. Brooks, *J. Am. Chem. Soc.,* 2004, **126**, 698–699.

[34] J. B. Klauda, R. M. Venable, J. A. Freites, J. W. O'Connor, D. J. Tobias, C. Mondragon-Ramirez, I. Vorobyov and A. D. MacKerell and R. W. Pastor, *J. Phys. Chem. B.,* 2010, **114**, 7830–7843.

[35] R. M. Venable, A. J. Sodt, B. Rogaski, H. Rui, E. Hatcher, A. D. MacKerell Jr, R. W. Pastor and J. B. Kauda, *Biophys. J.,* 2014, **107**, 134–145.

[36] W. Yu, X. He, K. Vanommeslaeghe and A. D. MacKerell, *J. Comput. Chem.,* 2012, **33**, 2451–2468.

[37] U. Essmann, L. Perera, M. L. Berkowitz, T. Darden, H. Lee and L. G. Pedersen, *J. Chem. Phys.,* 1995, **103**, 8577–8593.

[38] B. Hess, *J. Chem. Theory Comput.,* 2008, **4**, 116–122.

[39] S. Nosé, *Mol. Phys.,* 1984, **52**, 255–268.

[40] W. G. Hoover, *Phys. Rev. A.,* 1985, **31**, 1695–1697.

[41] M. Parrinello and A. Rahman, *J. Appl. Phys.,* 1981, **52**, 7182–7190.

[42] S. Nosé and M. L. Klein, *Mol. Phys.,* 1983, **50**, 1055–1076.
